# Coupling high-throughput mapping with proteomics analysis delineates *cis*-regulatory elements at high resolution

**DOI:** 10.1093/nar/gkab890

**Published:** 2021-10-11

**Authors:** Ting Wu, Danli Jiang, Meijuan Zou, Wei Sun, Di Wu, Jing Cui, Ian Huntress, Xinxia Peng, Gang Li

**Affiliations:** Aging Institute, University of Pittsburgh, Pittsburgh, PA 15219, USA; Department of Medicine, Xiangya School of Medicine, Central South University, Changsha 410083, China; Aging Institute, University of Pittsburgh, Pittsburgh, PA 15219, USA; Aging Institute, University of Pittsburgh, Pittsburgh, PA 15219, USA; Center for Pulmonary Vascular Biology and Medicine, Pittsburgh Heart, Lung, Blood, and Vascular Medicine Institute, University of Pittsburgh School of Medicine and University of Pittsburgh Medical Center, Pittsburgh, PA 15261, USA; Division of Oral Craniofacial Health Science, Adams School of Dentistry, Department of Biostatistics, UNC Gillings School of Global Public Health, University of North Carolina, NC 27599, USA; Department of Medicine, Division of Rheumatology, Immunology and Allergy, Brigham and Women's Hospital, Boston, MA 02115, USA; Department of Molecular Biomedical Sciences, North Carolina State University College of Veterinary Medicine, Raleigh, NC 27607, USA; Bioinformatics Graduate Program, North Carolina State University, Raleigh, NC 27695, USA; Bioinformatics Graduate Program, North Carolina State University, Raleigh, NC 27695, USA; Bioinformatics Research Center, North Carolina State University, Raleigh, NC 27695, USA; Aging Institute, University of Pittsburgh, Pittsburgh, PA 15219, USA; Department of Medicine, Division of Cardiology, University of Pittsburgh School of Medicine, Pittsburgh, PA 15223, USA

## Abstract

Growing evidence suggests that functional *cis*-regulatory elements (*cis*-REs) not only exist in epigenetically marked but also in unmarked sites of the human genome. While it is already difficult to identify *cis*-REs in the epigenetically marked sites, interrogating *cis*-REs residing within the unmarked sites is even more challenging. Here, we report adapting Reel-seq, an *in vitro* high-throughput (HTP) technique, to fine-map *cis*-REs at high resolution over a large region of the human genome in a systematic and continuous manner. Using Reel-seq, as a proof-of-principle, we identified 408 candidate *cis*-REs by mapping a 58 kb core region on the aging-related *CDKN2A/B* locus that harbors *p16^INK^^4^^a^*. By coupling Reel-seq with FREP-MS, a proteomics analysis technique, we characterized two *cis*-REs, one in an epigenetically marked site and the other in an epigenetically unmarked site. These elements are shown to regulate the *p16^INK^^4^^a^* expression over an ∼100 kb distance by recruiting the poly(A) binding protein PABPC1 and the transcription factor FOXC2. Downregulation of either PABPC1 or FOXC2 in human endothelial cells (ECs) can induce the *p16^INK^^4^^a^*-dependent cellular senescence. Thus, we confirmed the utility of Reel-seq and FREP-MS analyses for the systematic identification of *cis*-REs at high resolution over a large region of the human genome.

## INTRODUCTION

Gene transcription is primarily controlled by promoters that are typically located directly upstream or at the 5' end of the transcription initiation site. Transcription is also regulated by enhancers that activate promoter transcription over long distances. Both promoters and enhancers contain short DNA sequences that serve as *cis*-regulating elements (*cis*-REs) regulating gene transcription by recruiting regulatory proteins. In addition, transcription can also be regulated on the epigenetic level with heritable alterations that are not due to changes in the DNA sequence of the underlying *cis*-REs. Rather, epigenetic modifications, such as DNase hypersensitive sites, DNA methylation and histone modification, alter DNA accessibility by opening chromatin structure, thereby regulating gene transcription by making the underlying *cis*-REs accessible to regulatory proteins (1–5). Nevertheless, growing evidence also suggests that *cis*-REs exist in epigenetically unmarked sites and play unexpected roles in controlling gene transcription (6–8). Therefore, the ability to identify epigenetically marked and unmarked *cis*-REs in the human genome represents a yet unresolved challenge.

To date, a number of approaches have been developed to directly identify *cis*-REs. These include the use of comparative genomics to identify *cis*-REs with evolutionarily conserved sequences (9) and chromatin immunoprecipitation (ChIP) using specific antibodies to transcription factors to isolate *cis*-REs (10). Recently, high throughput methods have been developed for identifying *cis*-REs such as GRO/PRO-seq (11), CRISPRi-FlowFISH (12), CREST-seq (13), ATAC-seq (14), massive parallel reporter assay (MPRA) (15,16) and multiplexed editing regulatory assay (MERA) (6). While these techniques provide ways to identify *cis*-REs, they are, in general, technically complex to perform. In addition, some of these methods cannot be used to fine-map *cis*-REs with high resolution to a single *cis*-RE. Others identify *cis*-REs based on various epigenetic markers, making it difficult to use such methods to identify *cis*-REs in epigenetically unmarked region of the human genome. Thus, there remains an unmet need for complementary approaches that would enable a systematic and continuous mapping of *cis*-REs over a large region of the human genome regardless of epigenetic markers. Ideally, these approaches should also be amenable to a broad spectrum of cellular lineages, while employing widely accessible methods. Moreover, these approaches should identify *cis*-REs at high resolution to identify a single or double *cis*-REs so that each of the identified *cis*-REs could be confirmed and characterized using complementary techniques if needed.

*p16^INK4a^* is known as a tumor suppressor involved in the *p16*/cyclin-dependent kinase/retinoblastoma gene pathway of cell cycle control (17). Induction of the *p16^INK4a^* expression is considered as a marker of cellular senescence and accumulation of senescent cells in different tissues and organs is an important contributor to age-related diseases (18–25). However, despite its importance, few studies have been reported to elucidate the mechanisms that regulate the *p16^INK4a^* expression. Recently, epigenetic analysis has identified functional epigenetic marks on the *p16^INK4a^* promoter regions such as DNase hypersensitivity, histone modifications, and DNA methylation (26). Genome-wide association studies (GWAS) have also identified multiple single nucleotide polymorphisms (SNPs) that are associated with aging and age-related diseases on the *CDKN2A/B* locus that harbors *p16^INK4^^a^* as well as *p14^ARF^, p15^INK4^^b^*, and the antisense non-coding RNA in the CDK4 (INK4) locus (*ANRIL*) (27). In particular, GWAS have repeatedly identified a 58 kb core region at the *CDKN2A/B* locus strongly associated with atherosclerosis (28–30). This core region is ∼80 kb away from the transcription start site for *p16^INK4^^a^*. While both epigenetics and genetics provide the opportunity to facilitate the determination of *cis*-REs, identifying *cis*-REs in the regions that are not marked by any of these analyses on the *CDKN2A/B* locus is still a daunting task.

In this study, we report the adaptation of Reel-seq to systematically and continuously map *cis*-REs over a large region of the human genome at high resolution in a cell type-specific fashion. We used *p16^INK4^^a^* as a model system to evaluate this approach. By using Reel-seq, we screened the aging-related *CDKN2A/B* locus using the nuclear extract (NE) isolated from human primary endothelial cells (ECs) and identified 408 candidate *cis*-REs within the 58 kb core region. As a proof-of-principle, we performed an in-depth analysis with FREP-MS, a proteomics analysis technique (31), on two of these candidate *cis*-REs, either in an epigenetically marked or an unmarked site. We demonstrate that PABPC1 and FOXC2 are the regulatory proteins that specifically bind to these two *cis*-REs, thereby suppressing cellular senescence by downregulating the *p16^INK4a^* expression in human ECs. Thus, we demonstrate that sequential application of Reel-seq and FREP-MS can be utilized as a tool to systematically screen a large region of human genome to prioritize candidate *cis*-REs for further functional validation.

## MATERIALS AND METHODS

### Cell culture and reagents

Primary human arterial ECs (Cat#: CC-2535) were purchased from Lonza. ECs were free of mycoplasma and cultured in basal medium EGM-2 (Lonza) supplemented with 10% fetal bovine serum. All cells were cultured at 37°C in 5% CO_2_.

### Primers and antibodies

All primers used in this study were purchased from IDT and are listed in Supplementary Table S1. All antibodies used are listed in Supplementary Table S2 with the corresponding supplier information.

### Reel-seq for identification of *cis*-REs

To identify *cis*-REs, two Reel-seq libraries were created with the construct sequence shown in Supplementary Table S1. The two libraries were amplified and regenerated by primers New-seq and 926RR, or PE and G3, respectively, with Accuprime Taq polymerase (Invitrogen). For screening, ∼10 μg NE isolated from ECs was mixed with ∼50 ng of library DNA using the binding buffer from LightShift™ Chemiluminescent EMSA Kit (Thermo Fisher Scientific) and subsequently incubated at RT for 2 h. The reaction was performed in triplicate with three buffer-treated controls and three NE-treated samples. All samples were resolved on a 6% TBE native gel for gel shifting. After the completion of electrophoresis, unshifted bands from each of the controls and samples were cut and isolated by shaking incubation of the gel slices in TE buffer overnight. The isolated library DNA was next amplified by the same PCR amplification and the regenerated libraries were used for the next round of Reel-seq screen. In total, 10 rounds were performed. After the screening, next-generation sequencing (NGS) was performed with the PCR product from round 1, 4, 7 and 10.

### Definition of epigenetically marked and unmarked sites

Epigenetically marked sites are defined as regions that contain any of the following markers: H3K27ac, H3K4me1, H3K4me3, known transcription factor (TF)-binding sites, DNase I hypersensitivity sites, predicted DNase I hotspots or enhancers as predicted from chromatin modifications to defined DNA elements based on UCSC Genome Browser as well as the ENCODE database. We designated elements that do not contain any of these marks as an unmarked site.

### FREP-MS

FREP-MS assay was performed as previously described (31). The assay was performed in duplicate for each *cis*-RE with two negative controls and two samples. In brief, ∼10 μg of FREP construct DNA (Supplementary Table S1) was conjugated to 150 μl streptavidin-coupled Dynabeads (Life Technologies) according to the manufacturer's instructions. The DNA-beads were then washed and mixed with 1 mg NE isolated from human ECs cells at RT for 1 h. After separation and washing, the protein-DNA-beads were digested with 5 μl EcoR I (100 units/μl NEB) at 37°C for 30 min to remove the 3′ end DNA plus its binding proteins. After separation and washing, the protein–DNA-beads were subsequently digested with 5 μl BamH I (100 units/μl NEB) at 37°C for 45 min to release the *cis*-RE sequence plus its binding proteins. The supernatant was then collected for protein complex identification by mass spectrometry. To identify *cis*-RE-bound proteins, all those proteins that had peptide counts in both two samples and two controls were eliminated. *cis*-RE-bound proteins were identified as those that had peptide counts only in the two samples but not in the two controls.

### EMSA

EMSA was performed using the LightShift Chemiluminescent EMSA Kit (Thermo Fisher Scientific) according to the manufacturer's instructions. For the probe, a 35 bp DNA fragment containing a *cis*-RE was obtained by annealing two oligonucleotides. The double stranded oligonucleotides were then biotinylated using the Biotin 3' End DNA Labeling Kit (Thermo Fisher Scientific). After incubating the DNA with NE isolated from human ECs at RT for 30 min, the DNA-protein complex was resolved on a 6% TBE native gel for mobility shifting. The data represent three independent biological replicates (*n* = 3).

### Luciferase reporter assay

Luciferase reporter assay was performed in 293T cells using the reporter vector, pGL3-Promoter vector (cat#: E1761, Promega). The 35 bp S1606 and S961 fragments shown in Supplementary Table S1 were cloned into Sac I and Xho I sites in pGL3-Promoter vector individually (Supplementary Figure S1). For control, an irrelevant 35 bp fragment was cloned into the same vector. Constructs were transfected into 293T cells by FuGENE HD transfection reagent (Promega) together with an equal amount of the pRL-TK vector that provides constitutive expression of *Relilla* luciferase (Promega). The firefly luciferase reporter activity was normalizing to the *Relilla* luciferase activity using the Dual-Glo^®^ Luciferase Reporter Assay System (Promega). All experiments were performed according to the manufacturer's protocol. The data represent six independent biological replicates (*n* = 6).

### CRISPR/Cas9 gene editing

CRISPR/Cas9 gene editing was performed using the LentiCRISPR v2 vector system (Addgene, Plasmid #52961) with the gRNA sequences listed in Supplementary Table S1. Lentiviruses were infected into human Hela cells. Single puromycin-resistant clones were selected using limited-dilution cloning in 96-well plates. Genomic DNA was isolated from each clone and DNA fragments spanning S1606 and S961 were amplified and sequenced. Cells positive for mutations, except for the homozygous mutations, were subcloned and the same DNA fragments were cloned into TA vectors for sequencing of both alleles.

### ChIP assay

ChIP was performed as described previously (32). Briefly, the scrambled CRISPR/cas9 control Hela cells and the CRISPR/cas9 mutated Hela cells were cross-linked with 1% formaldehyde for 10 min. Sonication was carried out at 30% amplitude with 20 s on and 50 s off for 5 min. 10 μg anti-PABPC1 or anti-FOXC2 antibody coupled to Dynabeads™ Protein A/G (Thermo Fisher Scientific, Cat#:10001D and 10003D) was incubated with sonicated DNA at 4°C for overnight. Pulldown DNA was purified with Qiagen PCR purification kit after reversal of the crosslink. The purified DNA was used for real-time PCR analysis of the sequences around S1606 and S961 with primers S1606-ChIP-F/R and S961-ChIP-F/R (Supplementary Table S1). Rabbit IgG was used as an isotype control. The data represent the combination of three independent samples (*n* = 3).

### DNA-pulldown western blot analysis

DNA-pulldown western blot was performed as previously described (7). In brief, a 35 bp biotinylated DNA fragment was generated by annealing two biotinylated primers (IDT). Approximately 1 μg DNA was then attached to 40 μl of Dynabeads™ M-280 Streptavidin. DNA-beads were mixed with ∼100 μg of NE isolated from ECs at RT for 1 h with rotation. After washing off the unbound proteins, the DNA-bound proteins were eluted with sample buffer and resolved on an SDS-PAGE gel for western blot analysis using an antibody directed against PABPC1, FOXC2, MVP, DBN1, POLB or SERPINH1. For negative control, a 35 bp DNA fragment with irrelevant sequence was used. For an internal loading control, the same blot was probed using an antibody directed against PARP-1. The data represent three independent biological replicates (*n* = 3).

### qPCR analysis

Total RNA was isolated with the RNeasy Mini kit (Qiagen). 1 μg of RNA was digested with 1 U DNase I (Invitrogen) at 37°C for 30 min, followed by 65°C for 10 min after adding 1 μl 50 mM EDTA. cDNA was synthesized with SuperScript^®^ III Reverse Transcriptase (Invitrogen) according to manufacturers’ protocol. For qPCR, 50 ng cDNA was mixed with 0.5 μmol/l of each primer pair in a 20 μl total reaction using the Power SYBR Green PCR Master Mix (Applied Biosystems) or using the TaqMan Universal PCR Master Mix (Applied Biosystems). PCR amplification was carried out using an initial incubation at 94°C for 10 min, followed by 40 cycles of amplification step (94°C for 15 s, 60°C for 1 min), with the StepOne real-time PCR system. Data were analyzed using the ΔΔCT method with *GAPDH* as an endogenous reference. All the primers used are listed in Supplementary Table S1. The following probe/primer mixes for TaqMan PCR were purchased from Applied Biosystems: *p14^ARF^* (Cat#: Hs99999189_m1); *p15^INK4b^* (Cat#: Hs00793225_m1); *p16^INK4a^* (Cat#: Hs02902543_mH); *ANRIL* (Cat#: Hs04259472_m1) and *GAPDH* (Cat#: Hs02786624_g1). Data represent the combination of three independent samples (*n* = 3).

### Western blot analysis

Whole cell lysates were prepared using RIPA buffer (Sigma). Cytosolic proteins and nuclear proteins were isolated with NE-PER Nuclear and Cytoplasmic Extraction Reagents (Thermo Scientific) according to the manufacturer's instructions. Proteins were resolved on SDS-PAGE gels and transferred to PVDF membranes. Proteins were detected with gene-specific antibodies. All antibodies were purchased and used as listed in Supplementary Table S2. For a loading control, α-Tubulin was used. The data represent three independent biological replicates (*n* = 3).

### Senescence-associated (SA)-β-galactosidase Staining

The SA-β-Galactosidase Staining Kit (Cell Signaling, Danvers, MA, USA) was used to stain senescent ECs. The staining was visualized using an RVL-100-G microscope (Echo Laboratories, San Diego, CA, USA). Images were analyzed using ImageJ software (version 1.52K, NIH). The data represent three independent biological replicates (*n* = 3).

### γ-H2AX staining

Cells were plated on glass coverslips and fixed in 4% paraformaldehyde. For γ-H2AX staining, cell membranes were solubilized in PBS containing 5% FBS and 0.5% Triton X-100. Cells were first incubated with γ-H2AX antibodies in the solubilizing buffer for 1 h and immunofluorescence was detected with Alexa Fluor 488-conjugated secondary antibody. Cells were counterstained with 4′,6-diamidino-2-phenylindole (DAPI) (Sigma, Cat#:D9542). The staining was visualized using an RVL-100-G microscope (Echo Laboratories, San Diego, CA, USA). Images were analyzed using ImageJ software (version 1.52K, NIH). The data represent three independent biological replicates (*n* = 3).

### RNAi knockdown

For siRNA transient knockdown in human ECs, siRNAs for human FOXC2 and PABPC1 were purchased from Thermo Fisher (Cat #: 4427037 with ID: s194415 for FOXC2 and ID: s25664 for PABPC1) and knockdown was performed according to the manufacturer's protocol. For *FOXC2, PABPC1, MVP, DBN1, POLB* and *SERPINH1* shRNA knockdown in human ECs, lentiviruses were generated using the pLKO.1 puro vector (Addgene, Plasmid #8453). The targeted sequences are listed in Supplementary Table S1.

### Epigenetic analysis using Segway encyclopedia

To assess the potential association of candidate *cis-*REs with the local epigenetic states, we investigated the overlaps of candidate *cis*-REs and epigenetic annotations reported in the Segway encyclopedia (33). Since the Segway encyclopedia annotation was based on the previous human genome assembly version hg19, we first used the UCSC liftOver tool (default parameters minMatch = 0.95, minChainT = 0, minChainQ = 0, minBlocks = 1) to convert the genomic coordinates of Segway annotations onto the current genome assembly hg38. We then compared the overlaps of candidate *cis-*REs with two types of Seqway annotations. First, we used Segway's cell-specific epigenetic state classification which was a summary of the ENCODE ChIP-seq, DNase-seq and Repli-seq data used to build the Segway annotation. If a candidate *cis*-RE overlapped multiple Segway cell state classifications, we reported the Segway cell state classification with the longest overlap with the candidate *cis-*RE. The second annotation we used was Segway's cell type-agnostic encyclopedia of regulatory and transcriptional elements derived from the conservation-associated activity scores (CAASs). If a candidate cis-RE overlapped multiple Segway encyclopedia elements, we reported the average CAASs of all overlapping encyclopedia elements.

### Epigenetic analysis using ENCODE database

The UCSC genome browser (34) was used to visualize the data and create genomic view snapshots for *cis*-REs on the 58 kb core region at the *CDKN2A/B* locus.

#### Histone marker

The Layered H3K4Me1 and Layered H3K27Ac tracks show where modification of histone proteins is. We used the tracks of H3K4me1 and H3K27ac markers for all cell types listed in ENCODE.

#### DNase signal

We used the track of DNase I Hypersensitivity on all cell types listed in ENCODE.

#### GH Reg Elems

This track set contains regulatory elements, gene transcription starting sites, interactions between regulatory elements and genes, and clustered interactions.

#### ENCODE cCREs

This track displays the ENCODE Registry of candidate *cis*-regulatory elements in the human genome.

#### ORegAnno

This track displays literature-curated regulatory regions, transcription factor binding sites, and regulatory polymorphisms.

### Statistical analysis

For normally distributed data, all data were represented as standard error of mean (SEM). *P-*values were calculated using Student's *t* test with two tails.

## RESULTS

### Developing Reel-seq to identify *cis*-REs

**Figure 1.**
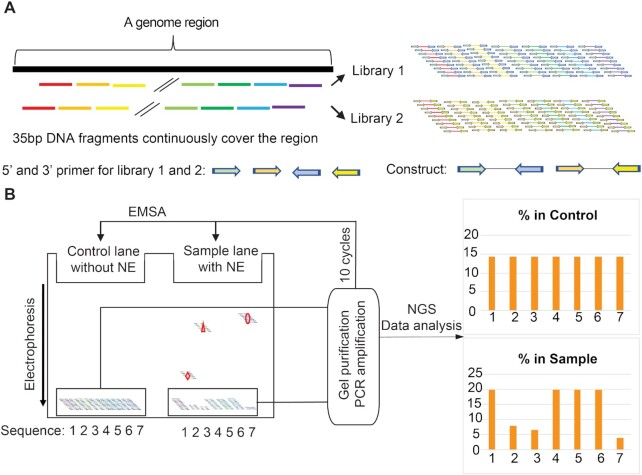
Reel-seq to identify *cis*-REs on the 58 kb core region within the *CDKN2A/B* locus. (**A**) Generation of Library 1 and 2 covering the entire region by overlapping fragments. (**B**) Simplified scheme showing to apply EMSA to identify *cis*-REs in an HTP fashion. Sequence 2, 3 and 7 have a decreased percentage in the sample pool compared to the control pool, suggesting they are candidate *cis*-REs. Non-specific shifting is not shown on EMSA, but it is considered to be evenly and randomly distributed. Red rhombus, triangle and oval representing nuclear proteins.

Previously, we introduced Reel-seq to identify functional SNPs (fSNPs) that are associated with diseases based on GWAS analysis in a high-throughput (HTP) fashion (7). Reel-seq is an electrophoresis mobility shift assay (EMSA)-based, unbiased HTP technique. It was originally designed to identify disease-associated fSNPs based on the fact that non-coding fSNPs influence risk gene expression by binding regulatory proteins (7) and that a typical transcription factor occupies ∼10 nucleotides with a range of 6–12 nucleotides (35,36). Therefore, by generating a synthetic DNA library that contains 31 bp SNP-centered DNA fragments, we were able to identify fSNPs in an HTP fashion (7). In this report, we sought to extend the utility of Reel-seq, allowing for the systematic identification of all *cis*-REs at high resolution over a large region of the human genome. For this, we re-designed the synthetic DNA library as shown in Figure 1A. In brief, a Reel-seq construct is engineered by placing a 35 bp DNA fragment between two primers. These primers are used for PCR amplification as well as for next generation sequencing. A Reel-seq library (e.g. Library 1) will be generated by hundreds of thousands of these constructs synthesized by massive parallel oligonucleotide synthesis to cover an entire DNA region. To cover the break points between each of two 35 bp fragments in Library 1, another Reel-seq library (e.g. Library 2) will be similarly generated. For screening, each Reel-seq library will be first mixed either with or without NE isolated from a particular cell type and to subsequently analyze the protein-DNA binding on a TBE native gel for gel shifting (Figure 1B, left). After electrophoresis, unshifted DNA libraries in both buffer-treated controls and NE-treated samples will be isolated and amplified by PCR. Amplified DNA libraries will then be used for another round of gel shift assay. After a total of 10 rounds of gel shifting, the percentage of each fragment in the buffer- and NE-treated libraries in different cycles will be quantified by next generation sequencing. If a DNA fragment is functional, it will be shifted on each round of the gel shift assay by its binding to regulatory proteins. As a result, the percentage of this DNA fragment in its library (unshifted) will decrease in the NE-treated samples compared to that in the buffer-treated controls (e.g. Sequence 2, 3 and 7 in Figure 1B, right). By using this approach, we should be able to rapidly identify *cis*-REs within a large region of the human genome in an HTP fashion.

### Reel-seq identifies *cis*-REs in the 58 kb core region on the *CDKN2A/B* locus associated with atherosclerosis

**Figure 2.**
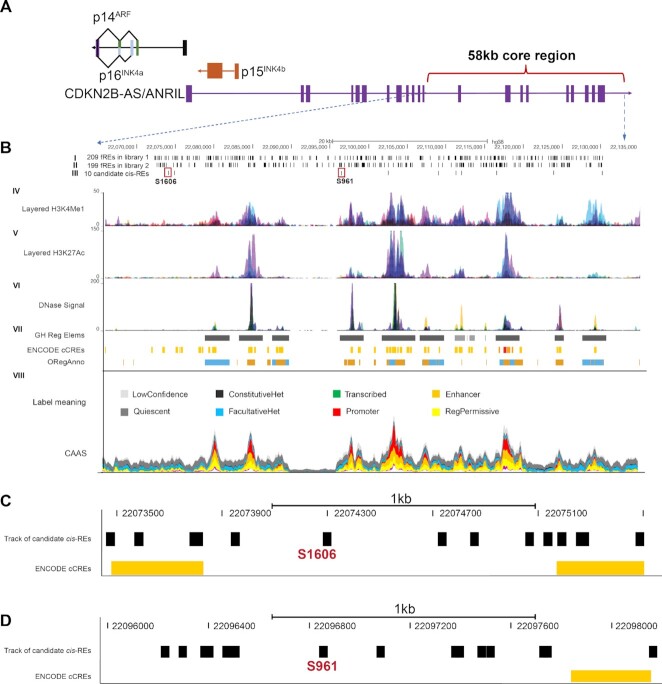
(**A**) The genomic view of the 58 kb core region on the *CDKN2A/B* locus. (**B**) Epigenetic analyses presenting different tracks. Track I and Track II show the location of the 408 candidate *cis*-REs identified by Reel-seq. Track III displays the 10 randomly picked *cis*-REs including S1606 and S961 indicated by the red square. Track IV to VI highlight the two epigenetic marks for H3K4me1, H3K27ac and DNase signal known as transcriptional factor-binding sites based on ChIP-seq data. Track VII shows the GH Reg Elems (genhancer regulatory elements and gen interactions), ENCODE cCREs (ENCODE candidate *cis*-regulatory elements combined from all cell types), and OregAnno (regulatory elements from OregAnno). Track VIII displays conservation-associated activity plot using Segway encyclopedia. The vertical axis indicates the conservation-associated activity score at a given position, colored proportionally to the fraction of the score that derives from each label type. CAAS: conservation-associated activity score. (**C**, **D**) a zoomed display showing the candidate *cis-*REs identified by Reel-seq (black) as well as three candidate enhancers predicted by ENCODE (orange) in the 2 kb region around S1606 and S961. ENCODE cCREs: candidate *cis*-Regulatory Elements.

To prove the feasibility of using Reel-seq to rapidly identify *cis*-REs at high resolution in an HTP manner, we chose to screen the 58 kb core region on the *CDKN2A/B* locus. A genomic view of the *CDKN2A/B* locus is diagramed in Figure 2A, which shows the four genes *p14^ARF^*, *p15^INK^^4^^b^*, *p16^INK4a^* and *ANRIL* located at this locus and the relative position of this 58 kb region located within the 3′ end of *ANRIL*. We are particularly interested in this 58 kb core region because GWAS have identified a strong association of this 58 kb region with different types of cardiovascular diseases including coronary artery disease (CAD), and all these diseases are recognized as age-related diseases since their incidence dramatically increases as a function of age (27). For the screening, we generated two synthetic DNA libraries using massive parallel oligonucleotide synthesis with one library containing 1669 and the other 1668 constructs, thus covering the entire 58 kb region. Of note, commercially synthesized DNA libraries can be used for screening immediately upon their receipt without any additional modification, which greatly simplifies the screening. The actual Reel-seq screen was carried out as outlined in Supplementary Figure S2A and the unshifted DNA libraries in both buffer-treated controls and NE-treated samples at round 1 were shown in Supplementary Figure S2B, which indicates about 50% of the DNA fragments are shifted in the three NE-treated samples by comparing to the three buffer-treated controls. The quality of this screen was evidenced by high reproducibility among the three repeats with all correlation coefficients demonstrating an *R*^2^ > 0.99 (Supplementary Figure S3). For the screening, NE isolated from primary human ECs was used as the source of regulatory proteins. In total, ten rounds of the gel shift assay were performed using three buffer-treated controls and three NE-treated samples. After the screen, for each library, 24 sub-libraries from rounds 1, 4, 7 and 10 (three buffer-treated controls and three NE-treated samples for each round) were recovered and prepared for NGS by incorporating 24 barcodes according to the standard NGS Illumina protocol. After sequencing, we recovered ∼1.1 × 10^8^ DNA fragments in each of the two libraries, among which we identified 4.1 × 10^7^ DNA fragments in Library 1 and 3.8 × 10^7^ in Library 2 with a sequence matching perfectly to their templates in the two libraries with a recovery rate of ∼35%.

To identify *cis*-REs, we first calculated the percentage of each 35 bp DNA fragment in each of the 24 sub-libraries and then calculated the average percentage for each DNA fragment from either the three NE-treated samples or the three buffer-treated controls in round 1, 4, 7 and 10. Using this averaged percentage, we further calculated the percentage ratio for each DNA fragment in the NE-treated samples versus the buffer-treated controls in round 1, 4, 7 and 10. We then applied these four ratios to calculate a slop*e*. By this, we sought to identify those fragments which exhibited a progressive decrease in their ratios across rounds 1, 4, 7 and 10 (slope < 0). Using this method, we identified a total of 1439 fragments in Library 1 and 1313 fragments in Library 2. Next, we calculated the *P-*value using a Student's t test on those fragments with a Slope <0 in round 1, 4, 7 and 10. Using this approach, we identified a total of 209 fragments in Library 1 and 199 fragments in Library 2 with a *P-*value < 0.05 in round 1, 4, 7 and 10 and a slope <0. We classified these 408 DNA fragments as candidate *cis*-REs. Among these 408 DNA fragments, there are 88 fragments that are overlapped between the two libraries. Based on a Pearson's Chi-squared test, this observed number of overlaps was statistically significant (*P-*value < 2.2E–16), and the expected number of overlapping fragments was 25. The detailed distribution of these 408 candidate *cis*-REs in the two libraries is present in the content of the 58 kb genomic region as shown in Figure 2B (Track I and II).

To compare the Reel-seq results with the existing data, we performed an *in sillico* analysis on the 58 kb core region at the *CDKN2A/B* locus using ENCODE database with all the cell types, including human endothelial cells, and conditions. In total, we identified 46 candidate regulatory regions including 45 predicted enhancers and one predicted promoter region and the average length of these regulatory regions is 271 bp (Supplementary Table S3). While six of these candidate enhancers do not contain any candidate *cis*-REs revealed by Reel-seq, 39 candidate enhancers and one promoter region are matched to the 100 candidate *cis*-REs as showing in Supplementary Table S4. Therefore, on average, each candidate enhancer could contain ∼2.5 candidate *cis*-REs, which indicates an enrichment of the 35 bp *cis*-REs in these 39 candidate enhancers. This result is consistent with the definition of an enhancer that could contain more than one transcription factor or regulatory protein binding sites (37,38). In addition, as we know that an enhancer can be 50–1500 bp long (39). If we take an enhancer as 140 bp long in general, which is equivalent to the length of four 35 bp fragments, we found that ∼82% our 408 candidate *cis*-REs can form a cluster with at least two *cis*-REs. We also performed epigenetic analysis on this 58 kb core region using Segway encyclopedia and the data are shown in Figure 2B (Track VIII) (33).

In addition, in Reel-seq screening, we want to keep as many positives as possible; therefore, we did not use any multiple testing adjustment for the *P-*value. In this case, we are aware of the probability of excessive false positives at the end of our data analysis using the Reel-seq screen. Therefore, downstream validation steps are always required to confirm these candidate *cis*-REs. Of note, due to the nature of Reel-seq screen that identifies candidate *cis*-REs in a defined 35 bp fragment, it is relatively easy to validate these candidate *cis*-REs by using contemporary techniques such as EMSA and luciferase reporter assay.

### Validation of *cis*-REs identified by Reel-seq

**Figure 3.**
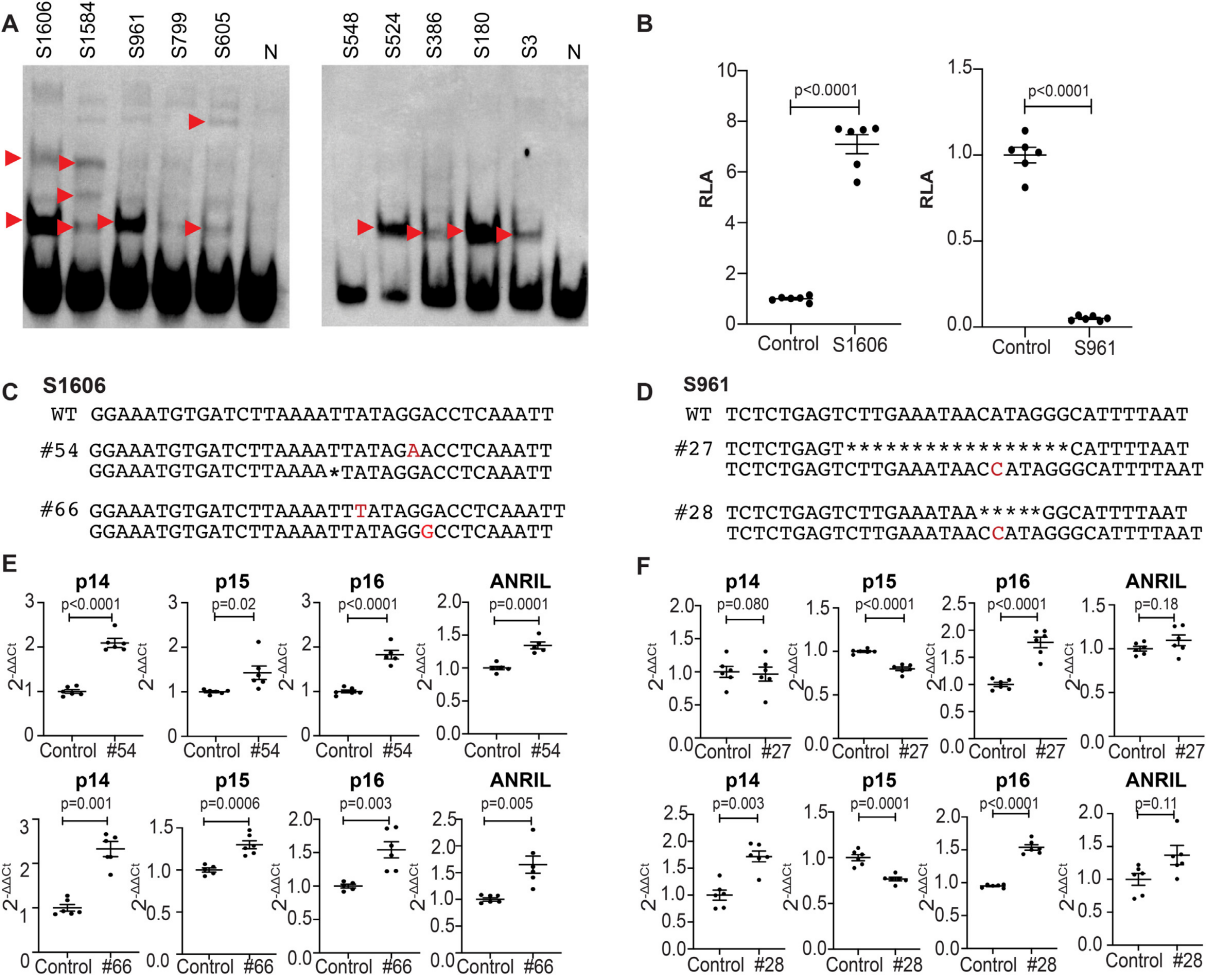
Validation of *cis*-REs identified by Reel-seq. (**A**) EMSA showing the gel shifting on 8 of the 10 candidate *cis*-REs identified in the Reel-seq screen on the *CDKN2A/B* locus. S799 in Library 1 and S548 in Library 2 show no shifted band, indicating they are false positives. S1606 and S961 in Library 1 were further analyzed. Red arrows indicate shifted bands. N: negative control. Data for EMSA represent three biologically independent samples (*n* = 3). (**B**) Luciferase reporter assay demonstrating the functionality of the *cis*-REs S1606 (left) and S961 (right). RLA: relative luciferase activity; Con: irrelevant sequence control; Data for luciferase reporter assay represents six biologically independent samples (*n* = 6). (**C**, **D**) Sequences showing mutations in two independent CRISPR/cas9 clones #54 and #66 on S1606 and clones #27 and #28 on S961 together with-wild type sequence. WT: wild-type. (**E**, **F**) qPCR showing the expression of *p14^ARF^, p15^INK4b^, p16^INK4a^* and *ANRIL* in clones #54 and #66 (left) and #27 and #28 (right). Ct: cycle threshold; Con: wild-type control. Data for qPCR analysis represent the combination of three biologically independent samples (*n* = 3), each performed in duplicate.

To demonstrate the success of the Reel-seq screening in identifying *cis*-REs, we performed an EMSA on ten randomly picked candidate *cis*-REs (five from each library). The position of these 10 candidate *cis*-REs is presented in Supplementary Table S5 as well as delineated in Track III of Figure 2B. Our results indicate that 8 of the 10 candidate *cis*-REs showed clear and unique EMSA bands (Figure 3A), suggesting a 20% false positive rate for this Reel-seq screening. We also randomly picked one non-functional fragment from each library identified by the same Reel-seq screen and performed an EMSA on these in parallel to the ten candidate *cis*-REs (Figure 3A). As expected, neither of these two fragments demonstrated any EMSA-specific bands. We also validated additional 19 candidate *cis*-REs that are not in the region of the candidate enhancers and promoters revealed by ENCODE by using EMSA and luciferase reporter assay. All these 19 candidate cis-REs were demonstrated as functional *cis*-REs showing a significant increase in the luciferase activities and a unique pattern of gel shifting in EMSA when compared to the negative control (Supplementary Figure S4). To confirm the functionality of these *cis*-REs, we selected S1606 and S961 in Library 1 for further analysis. S1606 is located ∼88 kb and S961 ∼102 kb away from the transcription start site of *p16^INK4a^* as shown in Figure 2B (Track III). Based on the analysis using UCSC genome browser, S1606 is located in an epigenetically unmarked region while S961 is in the middle of an enhancer (GH09J022096) in the Genehancer track (Figure 2B, Track VII: GH Reg Elems). We also performed *in sillico* analysis based on ENCODE database with all the cell types, including human endothelial cells, and conditions, we did not observe any existing *cis*-REs matched with neither S1606 nor S961 as shown in Figure 2C and D. To demonstrate that both S1606 and S961 are functional, we first performed a luciferase reporter assay. While an increase in luciferase activity was detected for S1606 (*P-*value < 0.0001) (Figure 3B, left), a decrease in luciferase reporter activity was observed for S961 (*P-*value < 0.0001) (Figure 3B, right). Next, CRISPR/cas9 gene editing was carried out using a gRNA that targets either S1606 or S961 in human HeLa cells. Two independent CRISPR-edited cell lines were obtained with mutations in each of these two elements. In clone #54 for S1606, there is a single nucleotide T deletion on one allele and a G-to-A transversion mutation on the other allele. In clone #66, there is a single nucleotide T insertion on one allele and an A-to-G transversion mutation on the other allele (Figure 3C). For S961, in both clones #27 and #28 there is a single nucleotide C insertion on one allele, and on the other allele, both clone #27 and clone #28 carry a deletion mutation of either 17 or 5 nucleotides, respectively (Figure 3D). To determine if these mutations change the expression of *p14*^ARF^*, p15*^INK^^4^^b^*, p16*^INK^^4^^a^ and *ANRIL*, the four putative effector genes located within the *CDKN2A/B* locus, a qPCR analysis was performed using total RNA isolated from these clones together with their controls. As a result, a significant upregulation of all four genes in clone #54 and #66 with mutations in S1606 was detected (Figure 3E). In contrast, mutations on S961 in clone #27 led to a significant downregulation of *p15^INK^^4^^b^* and an upregulation of *p16^INK^^4^^a^* without changing the expression of neither *p14^ARF^* nor *ANRIL* (Figure 3F, upper). Mutations in clone #28 showed a significant upregulation of the aforementioned putative effector genes except for *p15^INK^^4^^b^*, which was significantly downregulated as shown in Figure 3F (lower). These data, together with the data generated with EMSA (Figure 3A) and luciferase reporter assay (Figure 3B), demonstrate that both S1606 and S961 are *cis*-REs and mutations on these *cis*-REs can result in an altered expression of *p14^ARF^, p15^INK4b^, p16^INK4a^* and *ANRIL*. Additionally, these data demonstrate the utility of Reel-seq to identify *cis*-REs in an HTP fashion.

### FREP-MS identifies proteins that specifically bind to *cis*-REs on S1606 and S961

As *cis*-REs are fundamentally involved in gene expression regulation, their functionality relies on recruiting specific regulatory proteins including transcription factors to modulate gene expression. In order to determine the regulatory protein(s) that specifically bind to the *cis*-REs on S1606 and S961, we applied FREP-MS, a proteomics tool recently developed in our lab (31,40). FREP-MS is designed to identify proteins that specifically bind to a *cis*-RE by enzymatically separating the *cis*-RE together with its binding proteins from the remaining DNA-NE-bead complex in a DNA pulldown assay. Using NE isolated from human ECs in the FREP-MS, we identified five proteins, DBN1, POLB, MVP, PABPC1 and SERPINH1 specifically binding to S1606, and FOXC2 to S961, respectively, by mass spectrometry analysis (Table 1). While FOXC2 was previously reported to be a transcription factor that belongs to the forkhead box family containing a distinct DNA-binding forkhead box domain, all the five proteins that bind to S1606 have never been documented for acting as transcriptional regulators.

**Table 1.** Peptide spectrum counts showing proteins identified by FREP-MS binding to the *cis*-REs S1606 and S961

	Peptide spectrum count			
Protein ID	S1606		Control	
**DBN1**	12	3	0	0
**POLB**	4	3	0	0
**MVP**	3	3	0	0
**PABPC1**	6	2	0	0
**SERPINH1**	3	2	0	0
	**S961**		**Control**	
**FOXC2**	2	1	0	0

### Validation of the specific binding of PABPC1 and FOXC2 to *cis*-REs S1606 and S961, respectively

**Figure 4.**
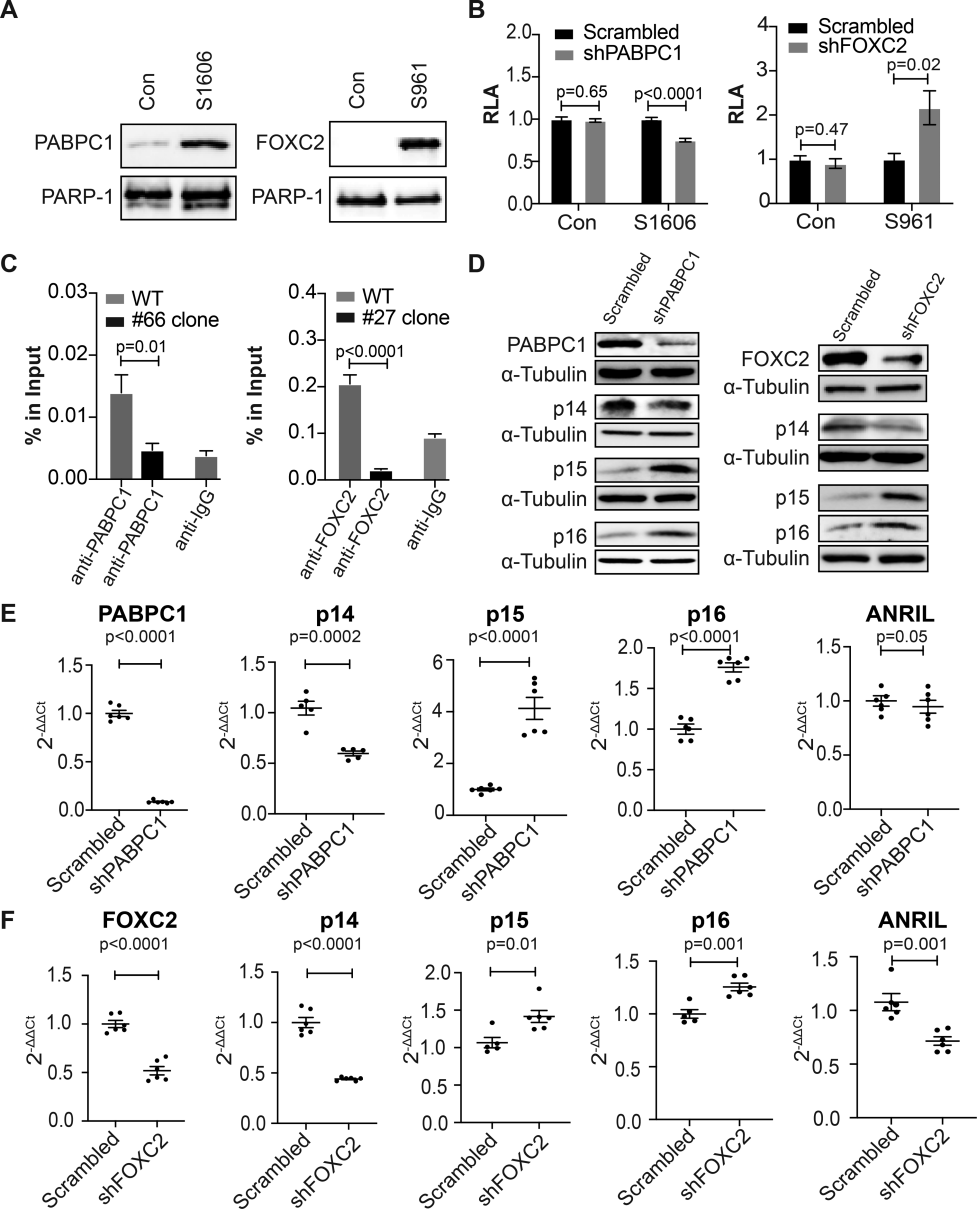
Demonstration of the specific binding of PABPC1 to S1606 and FOXC2 to S961. (**A**) DNA-pulldown western blots showing the specific binding of PABPC1 to S1606 (left) and FOXC2 to S961 (right). PARP-1 is used as an internal loading control. Con: an irrelevant sequence. Data for DNA pulldown western blot represent three biologically independent samples (*n* = 3). (**B**) Luciferase reporter assay using constructs containing either S1606 (left) or S961 (right) showing a decreased or an increased luciferase reporter activity in *PABPC1* (left) or *FOXC2* (right) shRNA knockdown T293 cells, respectively. RLA: relative luciferase activity; Con: an irrelevant sequence. The control for S1606 showed a scrambled level of luciferase reporter activity, however, this activity was not affected by shRNA knockdown of *PABPC1*. Data for luciferase reporter assay represent six biologically independent samples (*n* = 6). (**C**) ChIP assay demonstrating the specific binding of PABPC1 to S1606 (left) and FOXC2 to S961 (right). Data for ChIP assay represent three biologically independent experiments (*n* = 3). (**D)** Western blots showing an upregulation of *p16^INK4a^* and *p15^INK4b^* and a downregulation of *p14^ARF^* in the *PABPC1* (left) and *FOXC2* (right) shRNA knockdown human ECs. Scrambled: scrambled shRNA control. Data for western blot represent three biologically independent experiments (*n* = 3). (**E** and **F**). qPCR showing similar results in the regulation of *p14^ARF^, p15^INK4b^*, *p16^INK4a^* and *ANRIL* expression in the *PABPC1* (upper) and *FOXC2* (lower) shRNA knockdown human ECs. The expression of *ANRIL* is not significantly altered in the *PABPC1* shRNA knockdown human ECs but is significantly decreased in the *FOXC*2 shRNA knockdown human ECs. Data for qPCR analysis represent three biologically independent samples (*n* = 3), each performed in duplicate. sh: shRNA; Ct: cycle threshold.

To demonstrate the specific binding of these identified proteins to S1606 and S961, we further investigated the binding of PABPC1 to S1606 and FOXC2 to S961. First, we performed a DNA-pulldown western blot, an assay that is modified from AIDP-Wb, which is a novel DNA pulldown assay recently developed in our lab (7). Using this technique, we were able to verify the specific binding of FOXC2 to S961 and the highly enriched binding of PABPC1 to S1606, even though we did observe some level of nonspecific binding of PABPC1 to a control sequence (Figure 4A). Next, we performed a luciferase reporter assay with the luciferase reporter construct containing either S961 or S1606. We performed this assay in the setting of either *PABPC1* or *FOXC2* knockdown by shRNA. As indicated in Figure 4B, a significant decrease or increase in luciferase reporter activity was observed in either *PABPC1* or *FOXC2* shRNA knockdown cells. However, no difference in the luciferase reporter activity was observed when we performed the same assay using a control reporter construct containing an irrelevant sequence. Interestingly, in both the scrambled shRNA cells and the *PABPC1* shRNA knockdown cells, the level of luciferase reporter activity measured by using the control luciferase reporter construct is comparable to that shown in the scrambled shRNA control cells using the luciferase reporter construct carrying the S1606 sequence (Figure 4B left). These data suggest that there could be a regulatory factor other than PABPC1 that specifically binds to this control sequence. To further demonstrate the binding of PABPC1 to S1606 and FOXC2 to S961, we also performed a ChIP assay in the CRISPR-edited clone #66 for PABPC1 and clone #27 for FOXC2. We noted a significant enrichment of S1606 and S961 pulled down using either an anti-PABPC1-specific or an anti-FOXC2-specific antibody versus an anti-IgG antibody (Figure 4C). Using these two antibodies, we noted a significant decrease in the binding of PABPC1 to the mutated S1606 sequence (CRISPR-edited clone #66) and a similar decrease in the binding of FOXC2 to the mutated S961 sequence (CRISPR-edited clone #27) compared to wild-type controls (Figure 4C). In addition, we also performed an online search and identified the core binding motif of FOXC2 as TATGTAAATAA (41). This motif is highly similar to the sequence S961 TCTTGAAATAA, which is consistent with our epigenetic analysis showing that S961 is located in the middle of an enhancer (GH09J022096) (Figure 2B). Together, these data indicate that both PABPC1 and FOXC2 can specifically bind to S1606 and S961, respectively, thus demonstrating the fidelity of FREP-MS to identify proteins that specifically bind to *cis*-REs.

### PABPC1 and FOXC2 regulate the *p14^ARF^, p15^INK4b^, p16^INK4a^ and ANRIL* expression in primary human arterial ECs

Within the *CDKN2A/B* locus, there are three tumor suppressor genes: *p14^ARF^, p15^INK^^4^^b^* and *p16^INK^^4^^a^*, as well as *ANRIL*. To determine if these four genes are regulated by PABPC1 and FOXC2, we performed RNAi knockdown of *PABPC1* or *FOXC2* in primary human arterial ECs. Using a shRNA lentivirus that carries a shRNA sequence targeting either *PABPC1* or *FOXC2*, we were able to generate polyclonal pools of primary ECs having significantly reduced expression of either PABPC1 or FOXC2 as confirmed both by qPCR and western blot analysis (Figure 4D, E and F). In both the *PABPC1* and *FOXC2* shRNA knockdown ECs, we observed a significant upregulation of *p15^INK^^4^^b^* and *p16^INK^^4^^a^* expression whereas the expression of *p14^ARF^* was significantly downregulated on both the mRNA and protein levels (Figure 4D, E and F). The expression of *ANRIL* was unchanged in the *PABPC1* shRNA knockdown ECs but decreased in the *FOXC2* shRNA knockdown ECs (Figure 4E and F). To further confirm these data, we also performed RNAi knockdown in human arterial ECs using a siRNA that targets either *PABPC1* or *FOXC2* with a target sequence different from that employed in the shRNA knockdown. As a consequence, a similar result was observed in the expression of *p14^ARF^, p15^INK^^4^^b^, p16^INK4a^* and *ANRIL* as detected by qPCR (Supplementary Figure S5). Thus, these results demonstrate that PABPC1 and FOXC2 are transcriptional regulators modulating the expression of *p14^ARF^, p15^INK^^4^^b^, p16^INK^^4^^a^* and *ANRIL* in human arterial ECs.

### PABPC1 and FOXC2 suppress cellular senescence by downregulating the *p16*^*INK4a*^ expression

**Figure 5.**
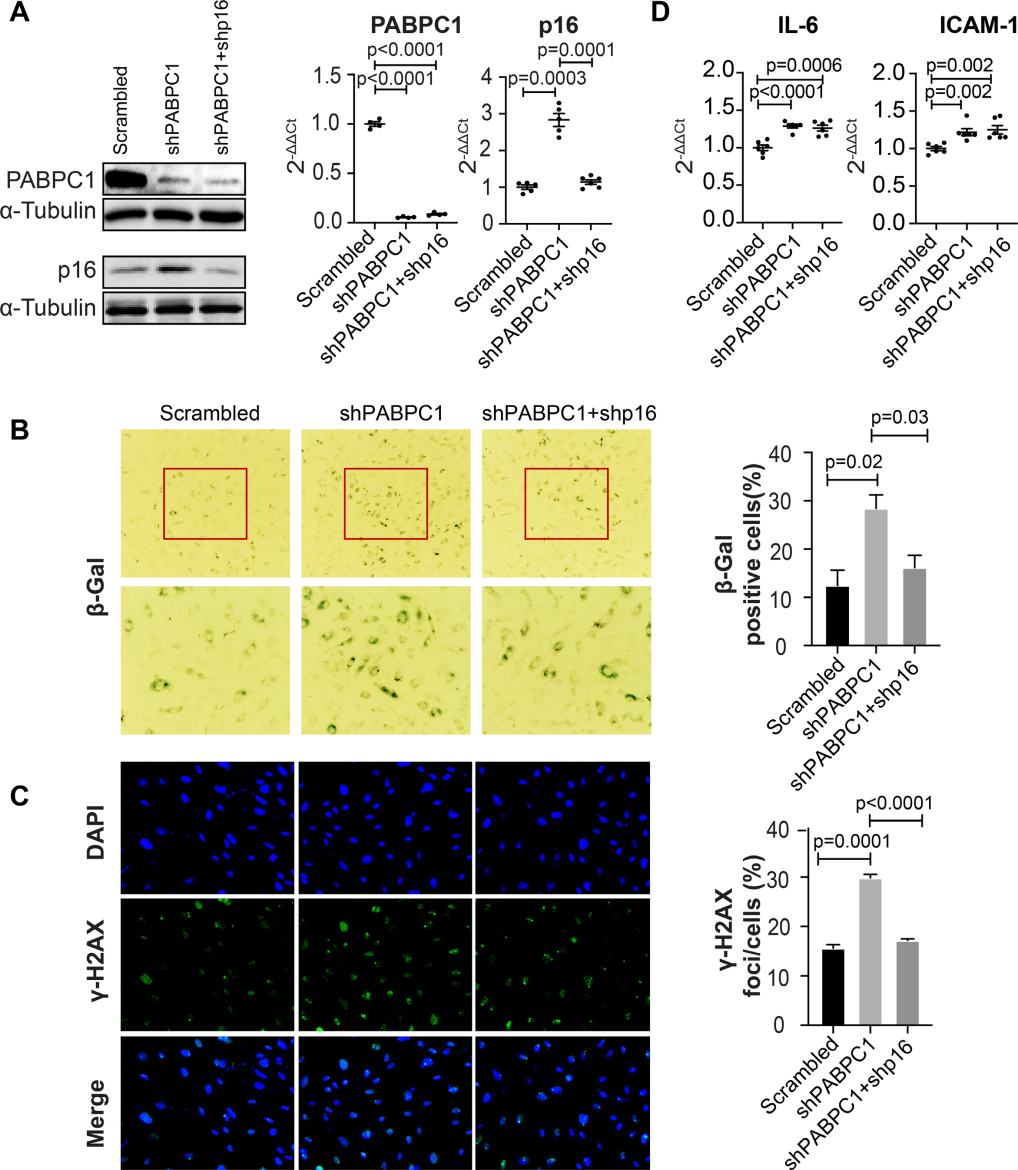
PABPC1 suppresses cellular senescence by downregulating the p16^INK4a^ expression. (**A**) Western blot (left panel) and qPCR analysis (right panel) showing an upregulation of *p16^INK4a^* in the *PABPC1* shRNA knockdown human ECs (middle lane) and a recovery of the *p16^INK4a^* expression in the *PABPC1* and *p16^INK4a^* double knockdown human ECs (right lane). Data for western blot represent three biologically independent experiments (*n* = 3). Data for qPCR analysis represent three biologically independent samples (*n* = 3), each performed in duplicate. (**B**) SA-β-gal and (**C**) γ-H2AX staining showing an increased cellular senescence in the *PABPC1* knockdown human ECs (middle panel) and a restoration of cellular senescence to the control level in the *PABPC1* and *p16^INK4a^* double knockdown human ECs (right panel). Quantitative analyses of the staining for both SA-β-gal and γ-H2AX are presented on the right side. Data for staining represent three biologically independent experiments (*n* = 3). (**D**) qPCR analysis showing an upregulation of *IL-6* (left panel) and *ICAM1* (right panel) in the *PABPC1* knockdown human ECs (middle lane). However, the upregulated expression of *IL-6* and *ICAM1* is not restored to the scrambled control level in the *PABPC1* and *p16^INK4a^* double knockdown human ECs (right lane). Ct: cycle threshold; sh: shRNA. Data for qPCR analysis represent three biologically independent samples (*n* = 3), each performed in duplicate.

**Figure 6.**
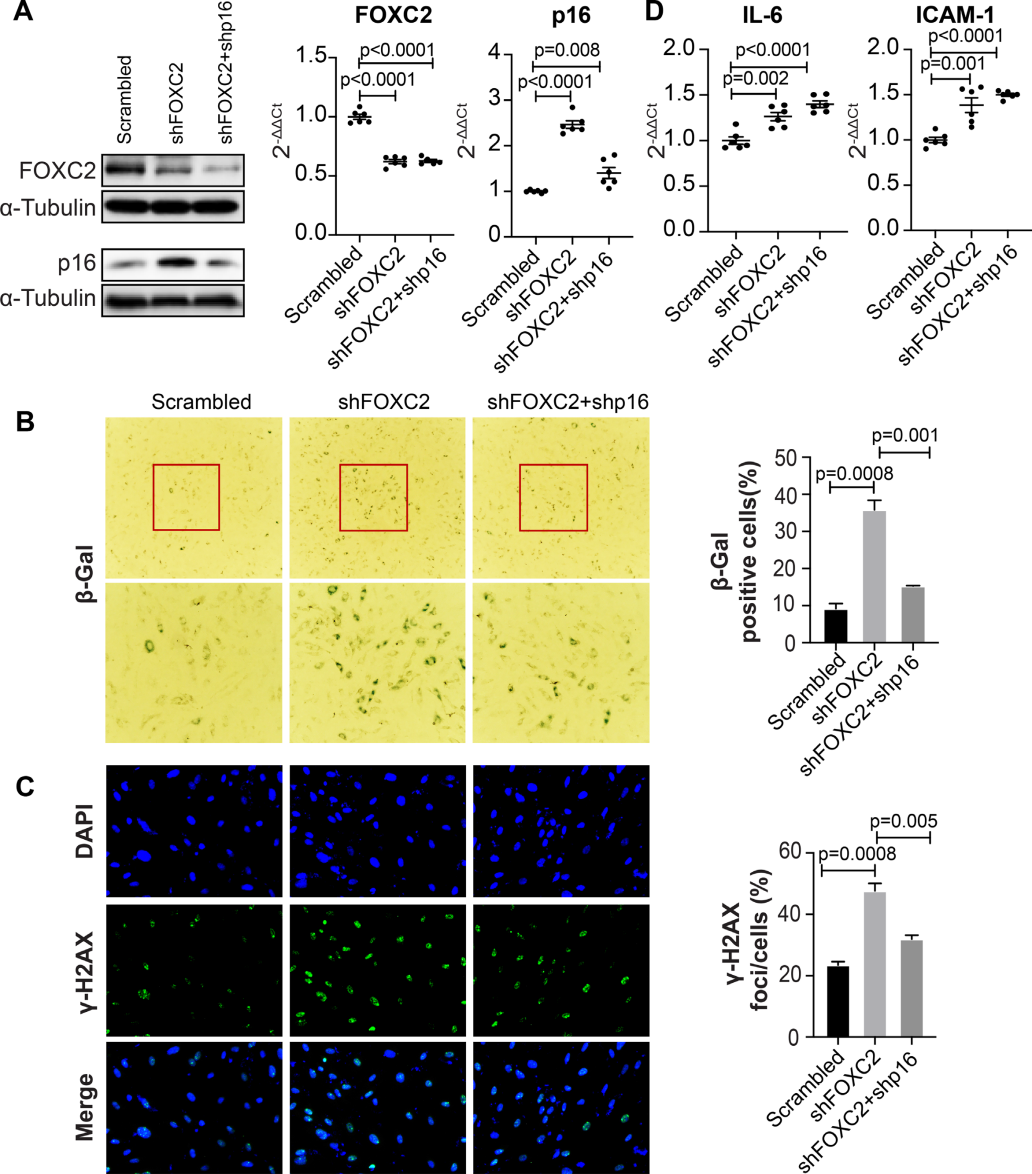
FOXC2 suppresses cellular senescence by downregulating the p16^INK4a^ expression. (**A**) Western blot (left panel) and qPCR analysis (right panel) showing an upregulation of *p16^INK4a^* in the F*OXC2* shRNA knockdown human ECs (middle lane) and a recovery of the p16^INK4a^ expression in the *FOXC2* and *p16^INK4a^* double knockdown human ECs (right lane). Data for western blots represent three biologically independent experiments (*n* = 3). Data for qPCR analysis represent three biologically independent samples (*n* = 3), each performed in duplicate. (**B**) SA-β-gal and (**C**) γ-H2AX staining showing an increased cellular senescence in the *FOXC2* knockdown human ECs (middle panel) and a restoration of cellular senescence to the control level in the *FOXC2* and p16^INK4a^ double knockdown human ECs (right panel). Quantitative analyses of both the SA-β-gal and γ-H2AX staining are presented on the right side. Data for staining represent three biologically independent experiments (*n* = 3). (**D**) qPCR analysis showing an upregulation of *IL-6* (left panel) and *ICAM1* (right panel) in the *FOXC2* knockdown human ECs (middle lane). However, the upregulated expression of *IL-6* and *ICAM1* is not restored to the scrambled control level in the *FOXC2* and *p16^INK4a^* double knockdown human ECs (right lane). Ct: cycle threshold; sh: shRNA. Data for qPCR analysis represent three biologically independent samples (*n* = 3), each performed in duplicate.

Among the four genes located in the CDKN2A/B locus, *p16^INK4a^* has been implicated in cellular senescence (18,19). Regulation of *p16^INK4a^* by both *PABPC1* and *FOXC2* in human arterial ECs suggests that both proteins might have important roles in endothelial senescence. To test this hypothesis, we first knocked down *PABPC1* in human ECs using shRNA lentiviruses. As predicted, an upregulation of *p16^INK4a^* induced by the downregulation of PABPC1 was demonstrated at both mRNA and protein levels (Figure 5A, left and middle lane). Consistent with the increased expression of *p16^INK4a^*, the *PABPC1* shRNA knockdown human ECs showed an increased level of cellular senescence as evidenced by both enhanced SA-β-gal (Figure 5B, left and middle panel) and γ-H2AX staining (Figure 5C, left and middle panel) as well as by the increased expression of the senescence-associated secretory phenotype (SASP) genes *IL-6* and *ICAM1* (Figure 5D, left and middle lane). To further demonstrate that downregulated PABPC1 activates cellular senescence through upregulating the *p16^INK4a^* expression, we inhibited the *p16^INK4a^* expression using a shRNA lentivirus in the *PABPC1* shRNA knockdown ECs. A shRNA-mediated downregulation of the *p16^INK4a^* expression was confirmed by both western blots and qPCR analysis (Figure 5A, right). Knockdown of *p16^INK4a^* in the *PABPC1* shRNA ECs restored cellular senescence to the same level as in the scrambled control ECs as determined by the SA-β-gal (Figure 5B, right panel) and γ-H2AX staining (Figure 5C, right panel). However, the expression of the SASP genes *IL-6* and *ICAM1* remained unchanged when *p16^INK4a^* was knocked down in the PABPC1 shRNA ECs (Figure 5D, right). This data recapitulates a previous observation showing that *p16^INK4a^* is not a SASP-inducing factor and that it can induce cellular senescence without the associated inflammatory secretory phenotypes (42). A similar result was obtained in the *FOXC2* shRNA knockdown human arterial ECs (Figure 6A–D). Again, knockdown of *FOXC2* resulted in a significant induction of cellular senescence in a *p16^INK4a^*-dependent fashion. Together, these data demonstrate that both PABPC1 and FOXC2 are suppressors of cellular senescence, and that downregulation of these two genes results in an upregulation of cellular senescence by increasing the *p16^INK4a^* expression.

### MVP, DBN1, POLB and SERPINH1 are suppressors of cellular senescence by inactivating *p16^INK4a^*

**Figure 7.**
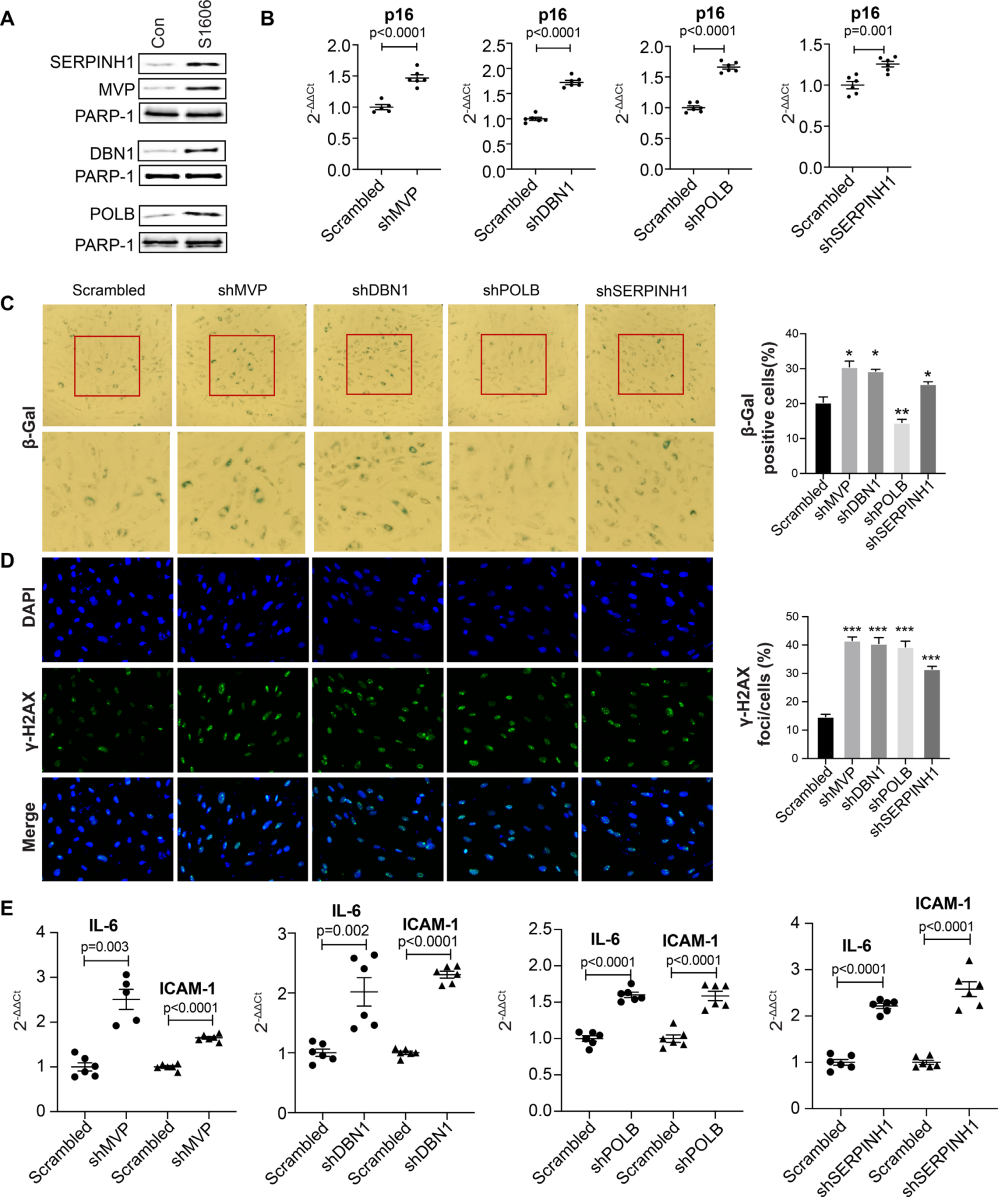
MVP, DBN1, POLB and SERPINH1 are suppressors of cellular senescence by inactivating the *p16^INK4a^* expression. (**A**) DNA-pulldown western blots showing the binding of MVP, DBN1, POLB and SERPINH1 to S1606. A trace amount of nonspecific binding of these proteins to the control DNA was detected. Con: an irrelevant sequence. Data for Western blots represent three biologically independent experiments (*n* = 3). (**B**) qPCR analysis showing a significant upregulation of the *p16*^*INK4a*^expression in the human ECs with either *MVP, DBN1, POLB* or *SERPINH1* knockdown by shRNA. Data for qPCR analysis represent three biologically independent samples (*n* = 3), each performed in duplicate. (**C**) SA-β-gal and (**D**) γ-H2AX staining showing an increased cellular senescence in the *MVP, DBN1, POLB* or *SERPINH1* shRNA knockdown human ECs. However, the SA-β-gal staining is decreased in the *POLB* knockdown human ECs. Quantitative analyses of both the SA-β-gal and g-H2AX staining are presented on the right side. * *P-*value < 0.05; **P-value < 0.01; and *** *P-*value < 0.001. Data for the staining represent three biologically independent experiments (*n* = 3). (**E**) qPCR analysis showing an upregulated expression of *IL-6* and *ICAM1* in the *MVP, DBN1, POLB* and *SERPINH1 shRNA* knockdown human ECs. Data for qPCR analysis represent three biologically independent samples (*n* = 3), each performed in duplicate. sh: shRNA.

Besides PABPC1, we also identified four other proteins, MVP, DBN1, POLB and SERPINH1 in the complex binding to S1606 (Table 1). None of these proteins were previously characterized as transcriptional regulators. To demonstrate that these four proteins are also regulators of cellular senescence via modulating the *p16^INK4a^* expression, we first performed DNA-pulldown western blots to validate the specific binding of these proteins to S1606 (Figure 7A). While an enriched binding of each of these four proteins to S1606 was observed, we also detected a trace amount of binding of these proteins to the negative control sequence (Figure 7A), similarly as observed for PABPC1 (Figure 4A). Next, we applied RNAi knockdown using shRNA lentiviruses to generate primary human ECs with downregulation of each of these four genes (Supplemental Figure S6). In each of these shRNA knockdown ECs, we observed a significant upregulation of the *p16^INK4a^* expression (Figure 7B). However, the expression of *p14^ARF^, p15^INK^^4^^b^* and *ANRIL* varied in these cells (Supplementary Figure S7). Except for POLB, consistent with the increased expression of *p16^INK4a^*, an increased cellular senescence was evidenced in these shRNA knockdown human ECs by both increased SA-β-gal (Figure 7C) and γ-H2AX (Figure 7D) staining. The SA-β-gal staining in the POLB shRNA knockdown ECs is significantly decreased instead (Figure 7C, the second from right). Additionally, the induction of cellular senescence in all these four shRNA knockdown ECs was also demonstrated by the significant increase in the expression of the SASP genes *IL-6* and *ICAM1* (Figure 7E).

## DISCUSSION

*cis*-REs are located in the non-coding regions of the human genome. Their identification is challenging, especially if they reside within epigenetically unmarked sites. One strategy to identify these elements is to develop a technique that can fine-map these *cis*-REs base-by-base, tiling a large region of the human genome in a systematic and continuous manner. However, considering the size of the human genome, such techniques have to be extremely simple in design and easy to perform. We believe that Reel-seq provides such a straightforward technique. We also believe that by coupling Reel-seq with FREP-MS, we will now be able to decipher the mechanisms of gene transcription regulation at a resolution that was not previously possible.

In this report, we demonstrate the feasibility and specificity of using Reel-seq by identifying *cis*-REs regulating the *p16^INK4a^* expression. Two such identified *cis*-REs were further characterized to regulate *p16^INK4a^*-dependent cellular senescence by recruiting PABPC1 and FOXC2. *FOXC2* is a transcription factor that belongs to the forkhead box family and participates in embryonic development (43). PABPC1 is a poly(A) binding protein that facilitates a variety of functions such as mRNA nuclear export, translation, and stability (44). So far, no data have reported any involvement of these two proteins in regulating the *p16^INK4a^* expression and cellular senescence. However, high level expression of FOXC2 and PABPC1 in gastric cancer (45,46) as well as in the activation of epithelial mesenchymal transition (EMT) and metastasis by overexpression of FOXC2 in cancer cells (47) are potentially consistent with their role in suppressing the *p16^INK4a^*-dependent senescence. Of note, it must be pointed out here that our data on FOXC2 and PABPC1 are preliminary and many questions remain to be answered. Therefore, further functional studies are required to demonstrate the precise role of FOXC2 and PABPC1 play in regulating the *p16^INK4a^* expression and cellular senescence.

We believe that there are several advantages in using Reel-seq and FREP-MS to identify and characterize *cis*-REs. Reel-seq is remarkably simple with HTP technology involving methods no more complex than standard PCR and gel shift assay. Unlike other HTP techniques such as MPRA (15,16) and MERA (6), Reel-seq does not need to create a DNA element reporter library or to express DNA element reporters in cells. Instead, commercially synthesized DNA oligonucleotide libraries can be directly used for screening immediately upon their receipt. Because of this simplicity and the low amount of NE used in gel shift assay, Reel-seq can be used as a prescreen using NE isolated from a range of disease-relevant cells/tissues and from different activation states to pinpoint the position of candidate *cis*-REs over a large region of the human genome and to prioritize these candidate *cis*-REs for further functional validation individually as we present in this study. FREP-MS is a remarkably efficient assay that usually takes only one day to perform. It is also a reliable technique to identify regulatory proteins, especially when we perform FREP-MS in a way that each *cis*-RE is assayed in duplicate so that we can increase the fidelity of mass spectrometry analysis and multiple *cis*-REs assays can be performed in parallel so to better compare them with each other, which will significantly increase the interpretation of their specificity (31,40).

We also recognize the disadvantage of using Reel-seq and FREP-MS. Unlike most *ex vivo* HTP reporter assays such as MPRA (15,16) and MERA (6), Reel-seq and FREP-MS are *in vitro* assays detecting the binding between a DNA fragment and regulatory protein(s) based on our knowledge that a typical transcription factor occupies 6–12 nucleotides (35). Therefore, they do not identify those elements that are involved in epigenetic regulation of transcription by modulating chromatin accessibility due to DNase hypersensitivity, histone modification or DNA methylation sites. Also, these techniques cannot reveal which gene(s) are actually regulated by a given *cis*-RE. Therefore, further functional analyses such as RNAi knockdown, ChIP assay, as well as CRISPR/cas9 gene editing are required to validate these *cis*-REs and their binding protein(s) in terms of their impact on regulating gene expression. Nonetheless, the strategy outlined here presents a simple, scalable approach to identify *cis*-REs contained within large genome regions of epigenetically marked or unmarked sites.

## DATA AVAILABILITY

All data are available in the supplementary files.

## Supplementary Material

gkab890_Supplemental_Files
