# Supplementary material for: Coupling high-throughput mapping with proteomics analysis delineates cis-regulatory elements at high resolution
Source: Nucleic Acids Res. 2021 Oct 11;50(1):e5. doi: 10.1093/nar/gkab890 (PMC8754656; doi:10.1093/nar/gkab890)
Supplement: gkab890_Supplemental_Files [file gkab890_supplemental_files.zip › Supplementary Figures and Tables.docx]

**Supplementary Figure 1**. A diagram showing the luciferase reporter construct pGL3-Promoter vector with the cloning sites Sac I and Xho I indicated.


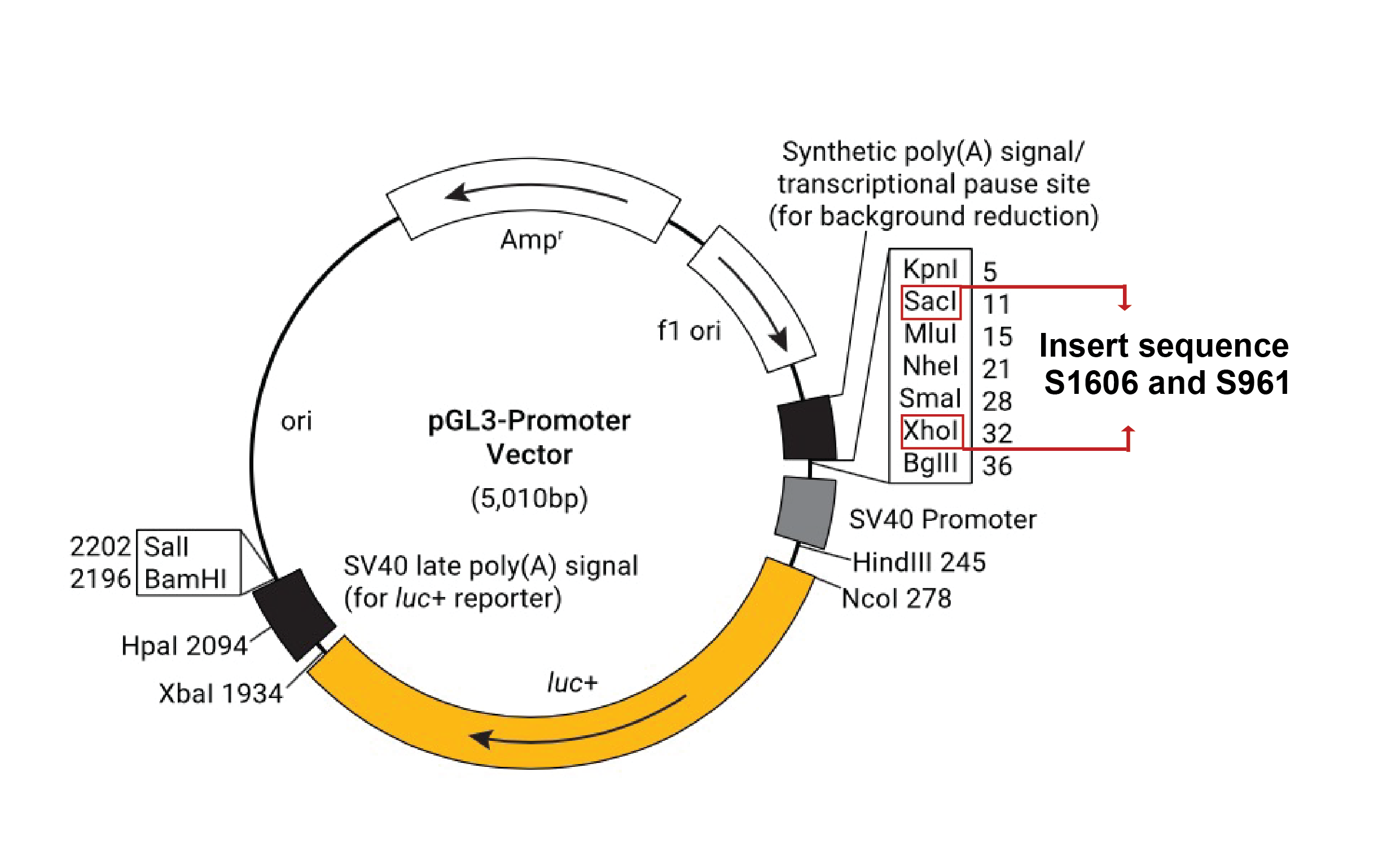


**Supplementary Figure 2. A.** Outline of the Reel-seq screen in the 58 kb core region on the *CDKN2A/B* locus. **B**. EMSA showing about ~50% of the DNA fragments are shifted in the three NE-treated samples by comparing to the three buffer-treated controls.

**
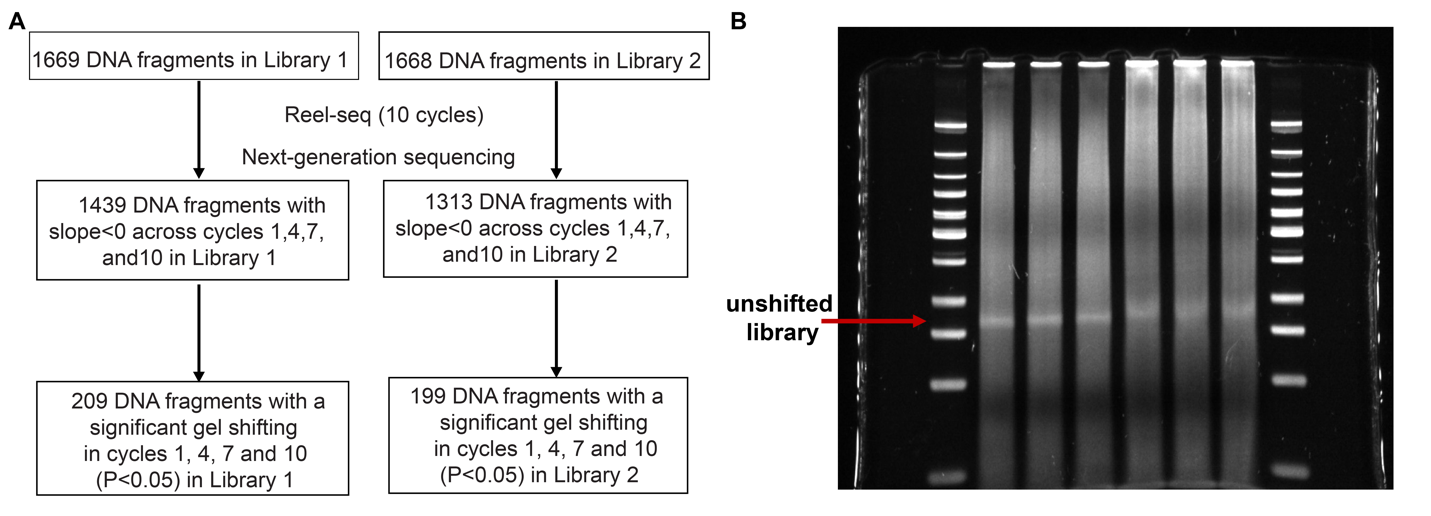
**

**Supplementary Figure 3.** Dot plots showing the high reproducibility of the Reel-seq screen with all correlation coefficients demonstrating an R^2^ > 0.99.


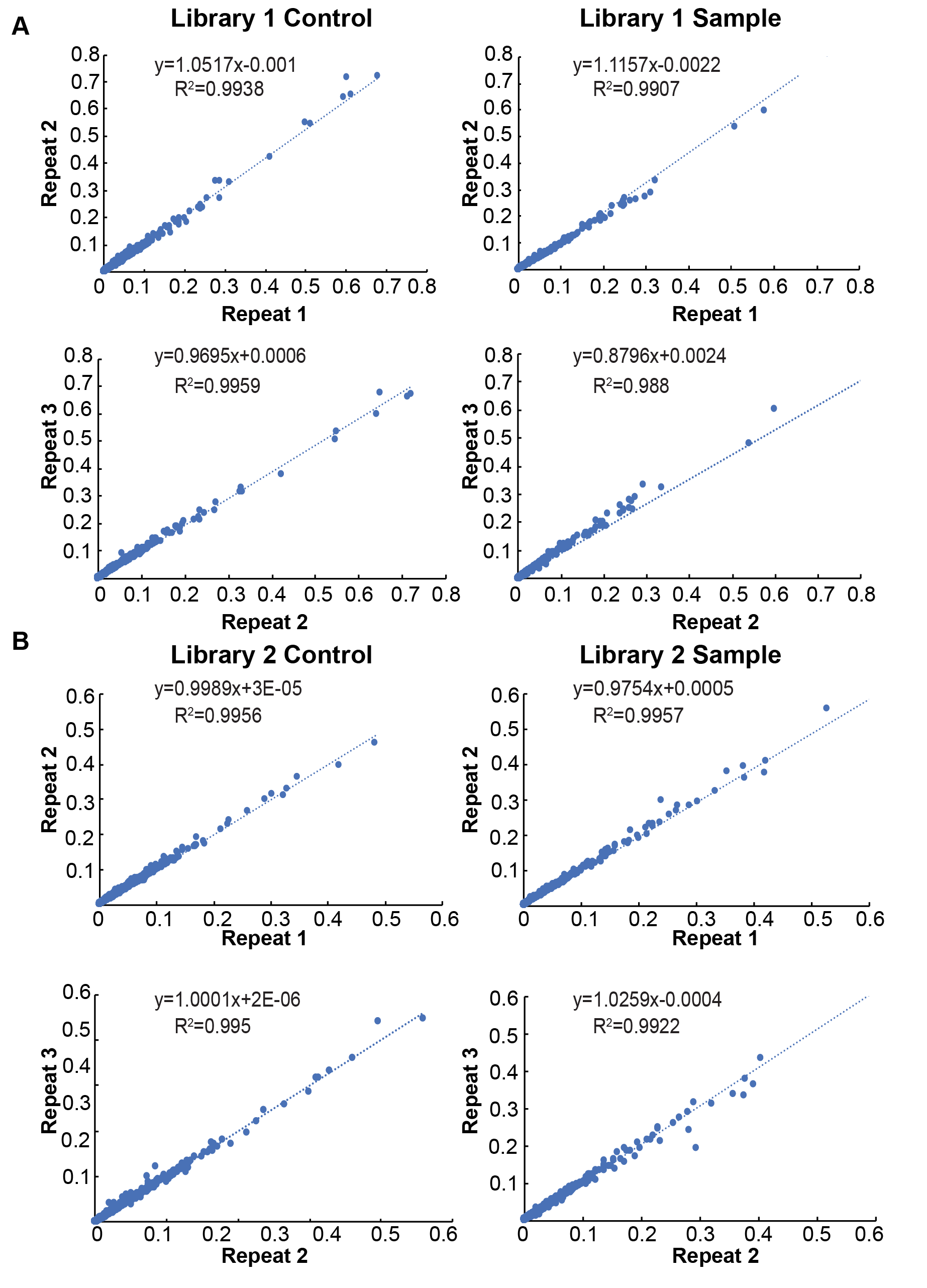


**Supplementary Figure 4. A.** Luciferase reporter assay and **B.** EMSA validating 19 *cis*-REs that are not in the region of the candidate enhancers and promoters revealed by ENCODE. pGL3: empty luciferase reporter construct; L2S93 (N): pGL3 with the negative fragment in the luciferase reporter assay and the negative fragment in EMSA. * P < 0.05; ** P < 0.001; and *** P , 0.0001.

**
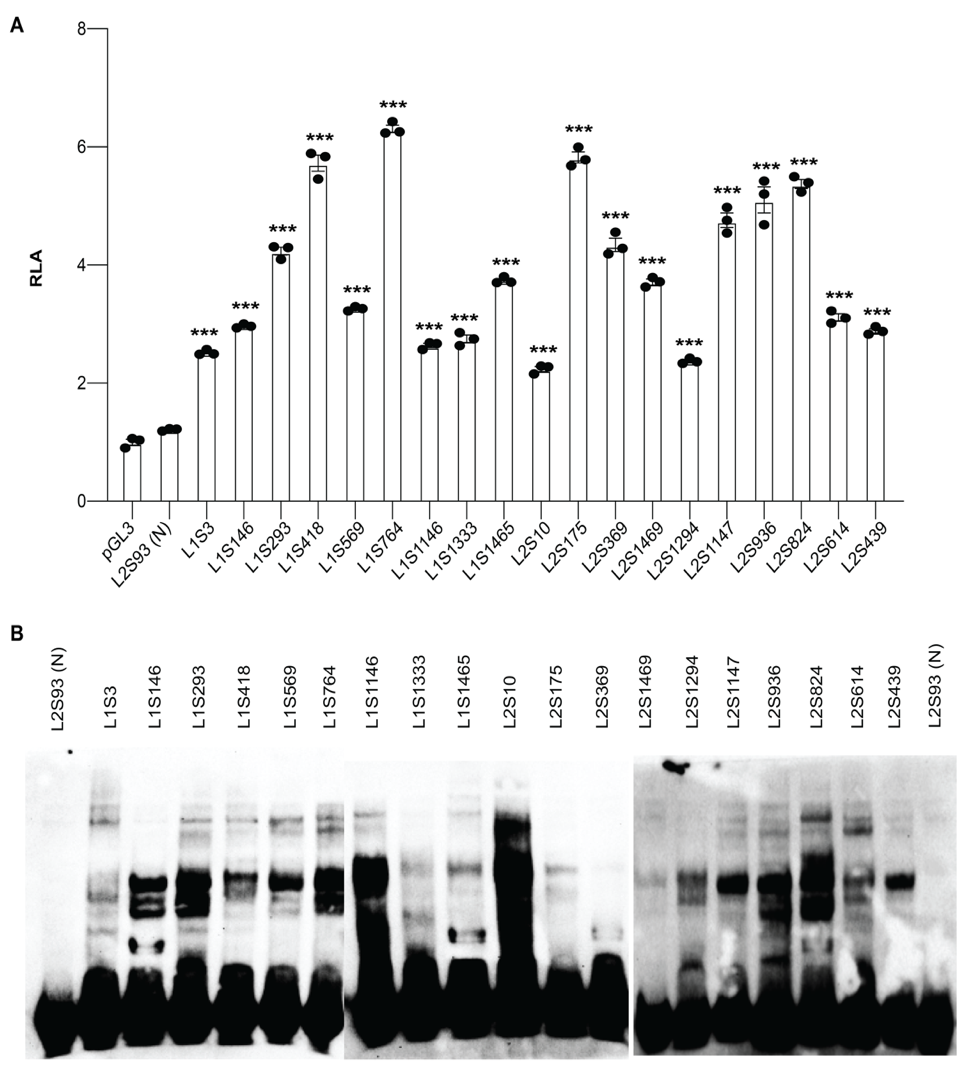
**

**Supplementary Figure 5. A** qPCR analysis showing the regulation of *p14^ARF^, p15^INK4b^*, *p16^INK4a^,* and *ANRIL* in the PABPC1 siRNA knockdown human ECs**. B.** qPCR analysis showing the regulation of *p14^ARF^, p15^INK4b^*, *p16^INK4a^,* and *ANRIL* in the FOXC2 siRNA knockdown human ECs**.**


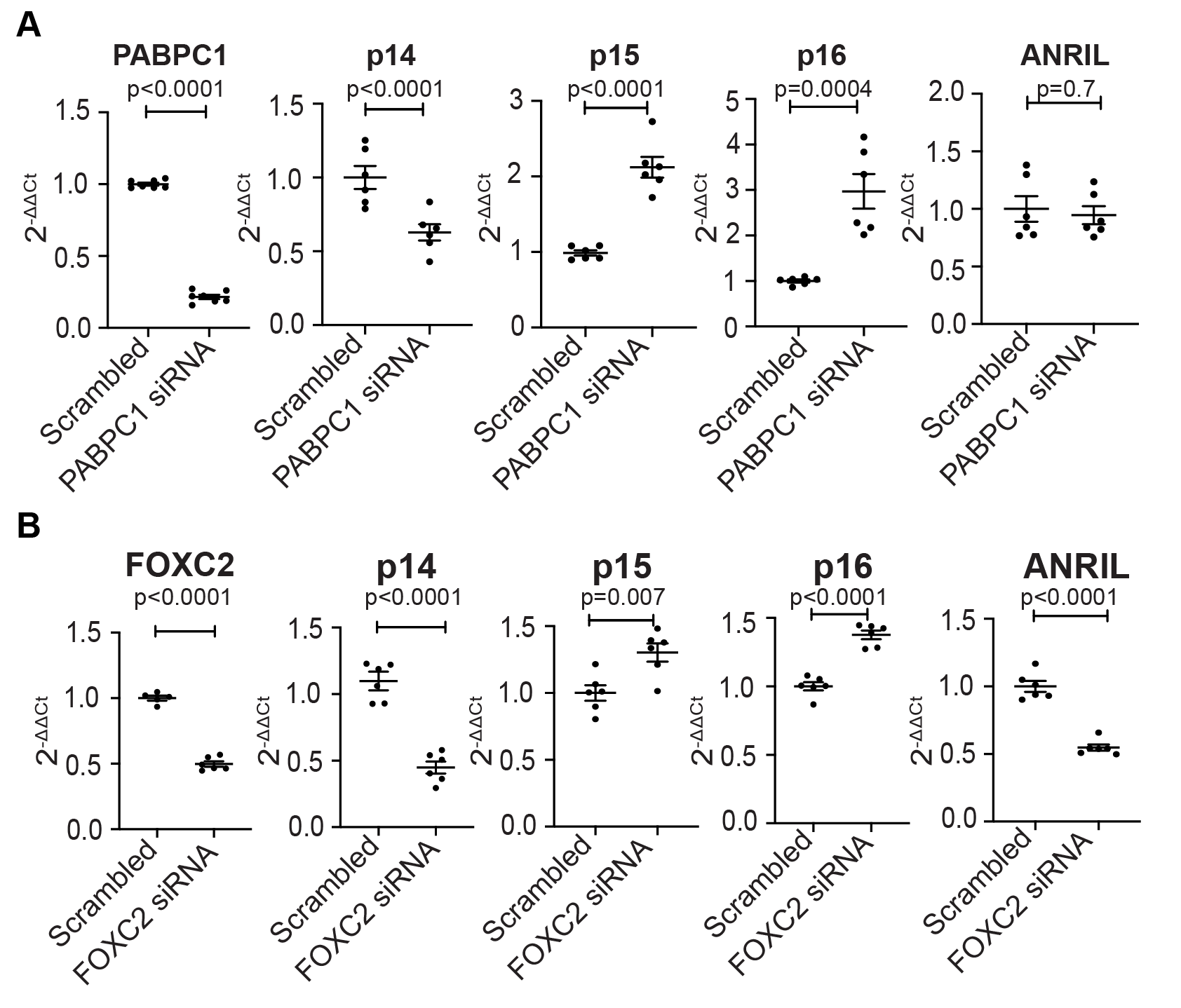


**Supplementary Figure 6.** qPCR analysis showing a downregulation of *MVP, DBN1, POLB*, and *SERPINH1* in the *MVP, DBN1, POLB,* or *SERPINH1* shRNA knockdown human ECs, respectively.


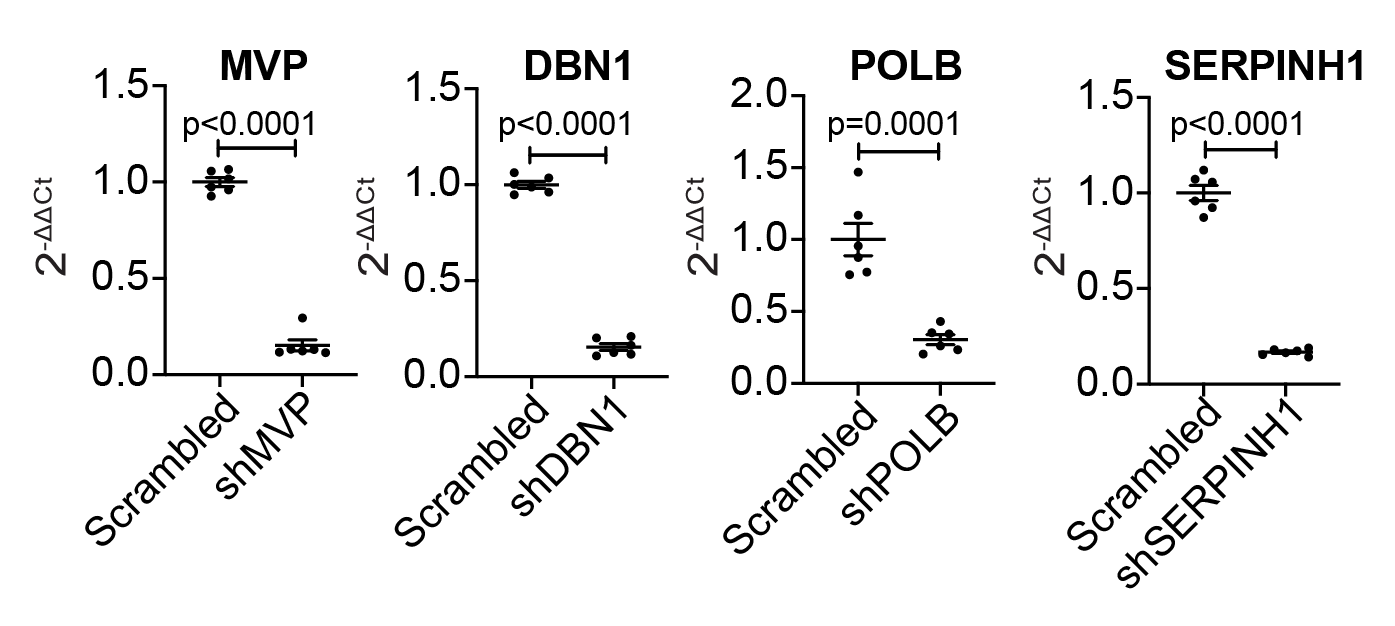


**Supplementary Figure 7. A-D.** qPCR analysis showing the regulation of *p14^ARF^, p15^INK4b^*, and *ANRIL* in the *MVP, DBN1, POLB*, or *SERPINH1* shRNA knockdown human ECs**,** respectively**.** Downregulation of *MVP, DBN1, POLB*, or *SERPINH1* by shRNA was demonstrated in **Supplementary Figure 6**.


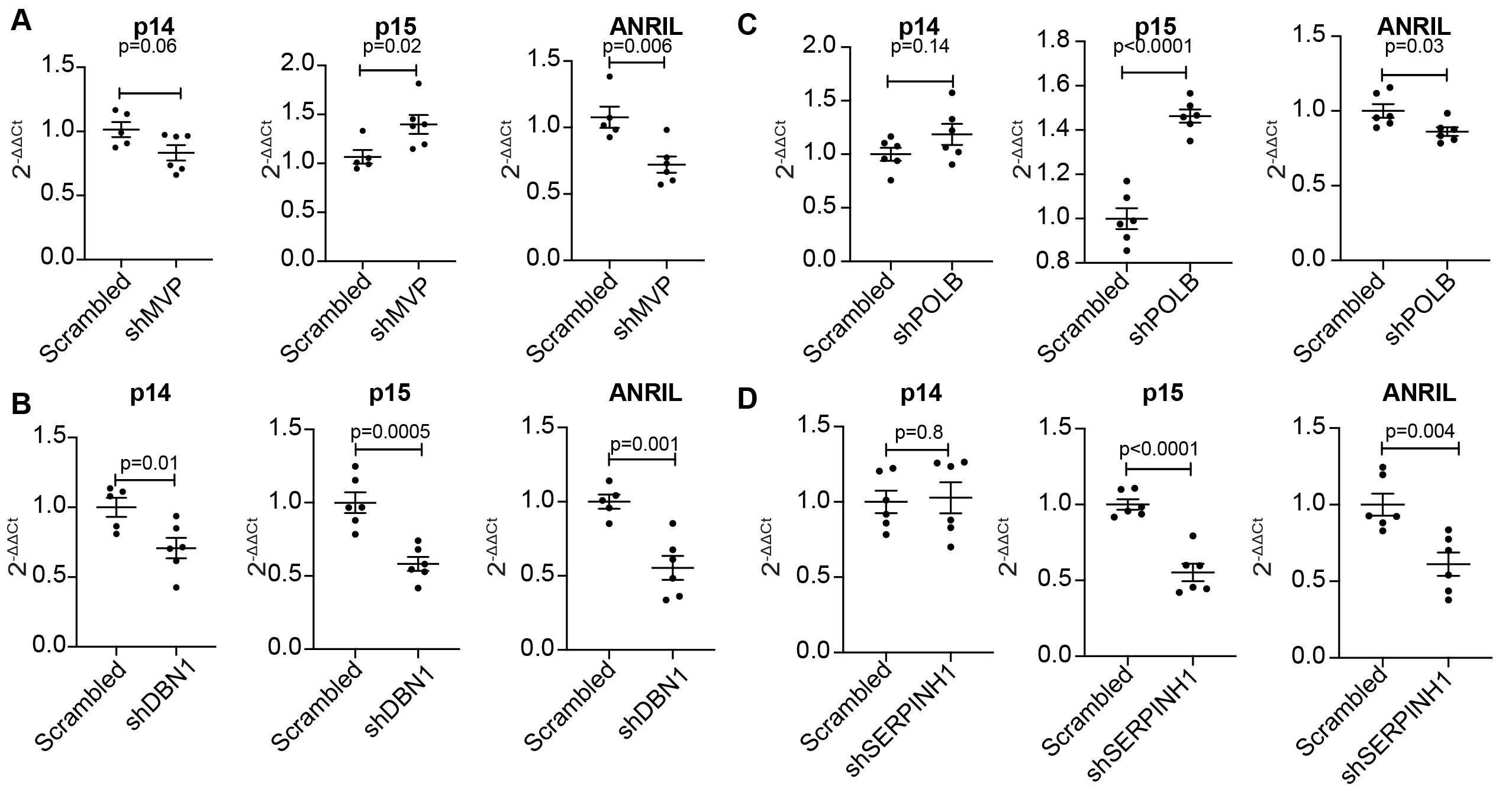


**Supplementary Table 1**. List of Primers used in this work

| **Usage** | **Primer name** | **Sequence** |
| --- | --- | --- |
| QPCR | IL6-F | GCAGAAAACAACCTGAACCTT |
|  | IL6-R | ACCTCAAACTCCAAAAGACCA |
|  | IL1b-F | ACAGATGAAGTGCTCCTTCCA |
|  | IL1b-R | GTCGGAGATTCGTAGCTGGAT |
|  | ICAM1-F | AGCGGCTGACGTGTGCAGTAAT |
|  | ICAM1-R | TCTGAGACCTCTGGCTTCGTCA |
|  | GAPDH-F | CGACCACTTTGTCAAGCTCA |
|  | GAPDH-R | AGGGGTCTACATGGCAACTG |
|  | PABPC1-F | AATCAACCCCTACCAGCCAG |
|  | PABPC1-R | CACGGTTCTGAGTCTGTGGG |
|  | FOXC2-F | GTAGGGGTCCCCATAGGTGT |
|  | FOXC2-R | TGTACAGCACGGTTGGAGAA |
|  | MVP-F | GTTTGATGTCACAGGGCAAGTTCG |
|  | MVP-R | CCTTTAGATGGAGGGCAGTGTTGG |
|  | SERPINH1-F | GCGCCTTGAAAAGCTGCTAA |
|  | SERPINH1-F | TTCTGCAGGTCATGGGTCAC |
|  | DBN1-F | AGGAGGAGTTTGCCCAATCG |
|  | DBN1-R | CGCTAATCACCACCCTCGAA |
|  | POLB-F | TTGGGAGTCACTGGAGTTGC |
|  | POLB-R | TGTGTCTGCCAGGGAGGATA |
| shRNA knockdown | PABPC1 shRNA | GCACCGTTCCACAGTATAAAT |
|  | FOXC2 shRNA | GCGGGAGATGTTCAACTCCCA |
|  | MVP shRNA | CCCATCAACCTCTTCAACACA |
|  | SERPINH1 shRNA | CCTCTACAACTACTACGACGA |
|  | DBN1 shRNA | CTGTGGAAATGAAGCGGATTA |
|  | POLB shRNA | CCTGTCAAAGGGTGAGACAAA |
| FREP | bioS1606-F | GTCTGTGTTCCGTTGTCCGTGCTGAATGGATCCGGATCCGGAAATGTGATCTTAAAATTATAGGACCTCAAATTGAATTCGAATTC |
|  | S1606-R | GAATTCGAATTCAATTTGAGGTCCTATAATTTTAAGATCACATTTCCGGATCCGGATCCA |
|  | bioS961-F | GTCTGTGTTCCGTTGTCCGTGCTGAATGGATCCGGATCCTCTCTGAGTCTTGAAATAACATAGGGCATTTTAATGAATTCGAATTC |
|  | S961-R | GAATTCGAATTCATTAAAATGCCCTATGTTATTTCAAGACTCAGAGAGGATCCGGATCCA |
|  | bio Negative-F | GTCTGTGTTCCGTTGTCCGTGCTGAATGGATCCGGATCCACCCGGAGGAACCACGGGGAAAGTGCGCTTCTGAGGAATTCGAATTC |
|  | Negative-R | GAATTCGAATTCCTCAGAAGCGCACTTTCCCCGTGGTTCCTCCGGGTGGATCCGGATCCA |
| Luciferase reporter | Luciferase reporter insert S1606 | GGAAATGTGATCTTAAAATTATAGGACCTCAAATT |
|  | Luciferase reporter insert S961 | TCTCTGAGTCTTGAAATAACATAGGGCATTTTAAT |
| CRISPR/CAS9 | gRNA-S1606 -F | CACCGAAATGTGATCTTAAAATTAT |
|  | gRNA-S1606 -R | AAACATAATTTTAAGATCACATTTC |
|  | gRNA-S961 -F | CACCGCTGAGTCTTGAAATAACATA |
|  | gRNA-S961 -R | AAACTATGTTATTTCAAGACTCAGC |
| Reel-seq | New-Seq | GGTGTGATGCTCGGATCCAGGAAC |
|  | 926RR | CACGAGACTACTGTCCTACC |
|  | PE | ACACGCACGATCCGACGGTAGTGT |
|  | G3 | CGAGCTTATCGTCGTCATCC |
| ChIP | S1606-ChIP-F | AGTTGCTCATTGTCCATGC |
|  | S1606-ChIP-R | TGTGCTTGGCACCTAGAGGATTTC |
|  | S961-ChIP-F | GATTGATGAGTGAGCTGGAGAAGG |
|  | S961-ChIP-R | CTGTCCCACTGGCATTCCATC |

**Supplementary Table 2**. List of Antibodies used in this work

| **Antibody** | **Manufacturer** | **Cat#** | **Usage** |
| --- | --- | --- | --- |
| FOXC2 | Invitrogen | 712371 | ChIP |
| FOXC2 | Proteintech | 23066-1-AP | WB |
| PABPC1 | Proteintech | 10970-1-AP | WB, ChIP |
| MVP | Proteintech | 16478-1-AP | WB |
| DBN1 | Proteintech | 10260-1-AP | WB |
| POLB | Proteintech | 18003-1-AP | WB |
| SERPINH1 | Proteintech | 10875-1-AP | WB |
| *p14^ARF^* | Invitrogen | MA5-14260 | WB |
| *p15^INK4b^* | Invitrogen | PA5-49749 | WB |
| *p16^INK4a^* | Proteintech | 10883-1-AP | WB |
| α-Tubulin | Sigma | T6074 | WB |
| PARP1 | Santa Cruz | Sc-7150 | WB |
| γ-H2AX | Santa Cruz | sc-517348 | IF |
| Alexa Fluor 488-conjugated antibodies | Invitrogen | A28175 | IF |

**Supplementary Table 3**. List of 45 predicted enhancer regions and one promoter region identified on the 58 kb core region at the CDKN2A/B locus using ENCODE database.

**Supplementary Table 4** (attached as a separate excel file). List of the sequence and position of the 408 candidate cis-REs on the 58 kb core region at the *CDKN2A/B* locus identified by Reel-seq and their corresponding results analyzed by using Segway encyclopedia and ENCODE database.

**Supplementary Table 5**. List of the sequence and position of the 10 candidate cis-REs assayed by EMSA as showing in **Fig. 3A**.

| **Name** | **Sequence** | **Chr** | **Start** | **End** |
| --- | --- | --- | --- | --- |
| S3 | GACAGAATTCTCTTTAAGTGGAAAATCAGCTTTAA | Chr9 | 22130286 | 22130320 |
| S180 | AATGTTCTAAATTGTTTGAGTAATATATGTTCATA | Chr9 | 22124091 | 22124125 |
| S605 | CCAGCTGATAGGGAAAGTAGGTATTATGGCTGAAC | Chr9 | 22109216 | 22109250 |
| S1584 | ATTTGTCTATGTACCATCTAAAATTCTCTCTTCCA | Chr9 | 22074951 | 22074985 |
| S1606 | GGAAATGTGATCTTAAAATTATAGGACCTCAAATT | Chr9 | 22074181 | 22074215 |
| S386 | TATTCCTTTTAGCCAAGTTATTCAGGAAGAATGGT | Chr9 | 22116864 | 22116898 |
| S524 | AAATTTTACTTTGTTTTCCTTTGAAAATACTTACT | Chr9 | 22112034 | 22112068 |
| S548 | TAGGCCTCAGTTTTGTAGTGCATCTTAAGATGAAC | Chr9 | 22111194 | 22111228 |
| S799 | TAGACCTTCTGCCTCCAAACTGTAAATACATTTCT | Chr9 | 22102409 | 22102443 |
| S961 | TCTCTGAGTCTTGAAATAACATAGGGCATTTTAAT | Chr9 | 22096739 | 22096773 |
